# Effect of fermented heat-treated rice bran on performance and possible role of intestinal microbiota in laying hens

**DOI:** 10.3389/fmicb.2023.1144567

**Published:** 2023-04-27

**Authors:** Yamei Wang, Weijiang Zheng, Wei Deng, Hua Fang, Heng Hu, He Zhu, Wen Yao

**Affiliations:** ^1^Department of Animal Nutrition and Feed Science, College of Animal Science and Technology, Nanjing Agricultural University, Nanjing, Jiangsu, China; ^2^Key Lab of Animal Physiology and Biochemistry, Ministry of Agriculture and Rural Affairs of the People’s Republic of China, Nanjing Agricultural University, Nanjing, Jiangsu, China

**Keywords:** laying hens, fermented heat-treated rice bran, apparent digestibility of nutrients, cecal microbiota, metabolic profiles

## Abstract

Rice bran is a high-quality and renewable livestock feed material rich in nutrients and bioactive substances. To investigate the effects of dietary supplementation with fermented heat-treated rice bran on the performance, apparent digestibility of nutrients, cecal microbiota and metabolites in laying hens, a total of 128 18-week-old Hy-Line brown layers were randomly assigned to four treatment groups: 2.5% HRB (basal diet contained 2.5% heat-treated rice bran), 5.0% HRB (5.0% heat-treated rice bran), 2.5% FHRB (2.5% fermented heat-treated rice bran), 5.0% FHRB (5.0% fermented heat-treated rice bran). Results showed that FHRB supplementation significantly increased the average daily feed intake (ADFI) during 25–28 weeks, and improved apparent digestibility of dry matter (DM), crude protein (CP), ether extract (EE) and crude fiber (CF) in laying hens. Moreover, feeding 5.0% of HRB and FHRB resulted higher egg production (EP) and average egg weight (AEW) during the feeding period, and decreased the feed conversion ratio (FCR) during 21 to 28 weeks. The alpha and beta diversity indices indicated that FHRB altered the cecal microbiota. In particular, dietary supplementation with FHRB significantly increased the relative abundances of *Lachnospira* and *Clostridium*. Compared with the 2.5% level of supplementation, supplementing 5.0% HRB and 5.0% FHRB increased the relative abundances of Firmicutes, *Ruminococcus* and *Peptococcus*, and lowered the relative abundance of Actinobacteria. Furthermore, dietary FHRB supplementation significantly increased the concentration of short-chain fatty acids in cecum and changed the overall metabolome. The results of correlation analysis showed a close interaction between cecal microbiota, metabolites and apparent digestibility of nutrients. Taken together, we revealed that FHRB supplementation can induce characteristic structural and metabolic changes in the cecal microbiome, which could potentially promote nutrient digestion and absorption, and improve the production performance of laying hens.

## Introduction

Rice bran is a major byproduct produced during rice processing and comprises approximately 5%–12% of the weight of brown rice (Ti et al., 2015). It contains more than 64% of the nutrients in brown rice and is rich in proteins, fats, vitamins and bioactive components, such as dietary fiber, oryzanol, flavonoids and phenols with high biological potentials and low allergenicity (Schmidt et al., 2014; Le et al., 2019). However, fresh rice bran has poor storage stability due to its high oil content and contains antinutritional factors, such as phytic acid and trypsin inhibitor (Wu et al., 2020), which limit its application in animal feed and lead to the waste of resources and environmental pollution (Kim et al., 2018). Therefore, it should be treated with stabilization before utilization. Heat treatment, which can inactivate lipase and oxidase and reduce antinutritional factors to improve storage stability, is the most commonly used pretreatment method for fresh rice bran (Wanyo et al., 2016; Yang et al., 2021). The probiotic fermentation of unconventional feed can reduce antinutritional factors and improve the digestibility of feed. On the one hand, microorganisms produce a series of enzymes during the fermentation process, which can decompose macromolecular substances into low-molecular-weight substances and promote the absorption and utilization of nutrients by animals (Li et al., 2020). Moreover, fermented unconventional feed contains a large number of probiotics and their metabolites, which can stabilize the dominant intestinal flora, inhibit the growth of harmful bacteria, maintain the balance of intestinal microecology, and improve intestinal health and production performance (Guo et al., 2021). Due to the high oil content in fresh rice bran, which is easily hydrolyzed and oxidized to produce peroxides under the action of inherent lipases and lipoxygenases. These compounds are further oxidized to produce aldehydes, ketones and other oxidative substances, undergo oxidative rancidity and release off-flavors (Malekian et al., 2000). We obtained a product patent of stably fermented rice bran combining heat treatment and probiotic fermentation to improve the feeding value of rice bran (CN 114946998 A). Probiotic metabolism during the fermentation of rice bran produces several enzymes and other beneficial components to reduce levels of antinutritional factors, which may have potential health-promoting properties (Seyoum et al., 2022; Su et al., 2022).

The risk of intestinal bacterial diseases and microbial imbalances increases in laying hens due to the complete restriction on antibiotics in feed (Paiva and Mcelroy, 2014). As a result, alternatives, such as microecological agents, enzyme preparations, and fermented feed, are constantly being developed. Fermented feed can fully utilize unconventional feed to compensate for feed material shortages and enhance the culture environment, both of which are crucial for the breeding of laying hens without the use of antibiotics. Fermented feed has a certain favorable impact on the intestinal flora and host health (Guo et al., 2021), increases the amount of lactic acid bacteria and the concentration of SCFAs in feces, improves intestinal digestive and metabolic activities, promotes antioxidant and anti-inflammatory effects, and enhances the production performance and egg quality of laying hens (Huang et al., 2020; Li et al., 2020; Zhu et al., 2022). The gastrointestinal tract is a complex environment (Kelly and Conway, 2001; Wei et al., 2013), and both the feeding environment and its health status affect the composition of intestinal microbes (Wilson et al., 2005). The cecum in particular is responsible for further nutritional absorption, microbial fermentation, and hazardous chemical degradation (Gong et al., 2002). The cecal microbiota plays a key role in which, and helping to resist the colonization of invasive pathogens (Pandit et al., 2018). Gut microbial function is maintained by probiotics and their metabolites, such as SCFAs, organic acids, vitamins, and other metabolites (Nagpal et al., 2018; Zielinska and Kolozyn-Krajewska, 2018; Oviedo-Rondon, 2019; Yao et al., 2022).

Therefore, our study aims to investigate the effects of fermented heat-treated rice bran (FHRB) on the laying performance, apparent digestibility of nutrients, cecal microbiota and metabolites of laying hens by adding different levels of FHRB to the diet, which may provide a theoretical basis for the application of rice bran and its fermented products in laying hens.

## Materials and methods

### Fermentation of heat-treated rice bran with *Bacillus subtilis* and *Lactobacillus plantarum*

Fresh rice bran was oven dried at 120°C for 30 min in a blast drying oven, cooled to room temperature, and stored at 4°C till further use. Conditions for HRB fermentation were optimized using orthogonal tests with free radical scavenging rate as an index parameter. Fermentation of HRB was performed for 36 h at 35°C using 0.8‰ mixed inoculum containing *Bacillus subtilis* and *Lactobacillus plantarum* in a ratio of 2:8 and HRB to water ratio of 1:0.6. The HRB and FHRB were evaluated for presence mycotoxins and microbial pollutants to meet the General Administration of Quality Supervision et al. (2018). Among them, the orthogonal test results were provided in supplementary file (Supplementary Table S1).

### Animals, experimental design, and sampling

A total of 128 Hy-Line Brown laying hens (18 weeks old) were randomly assigned to four groups. Each group had eight replicates with four birds per replicate. A 2 × 2 factorial design was used to investigate the effects of probiotic fermentation (HRB or FHRB), feeding level (2.5% or 5.0%) and their interactions on laying hens. The treatments were as follows: (1) 2.5% heat-treated rice bran (2.5% HRB); (2) 5.0% heat-treated rice bran (5.0% HRB); (3) 2.5% fermented heat-treated rice bran (2.5% FHRB); and (4) 5.0% fermented heat-treated rice bran (5.0% FHRB).

All experimental birds were housed in multilayer cages with adequate ventilation and a controlled environment with a 16-h light/8-h dark cycle. The room temperature was maintained at 23 ± 2°C, and the relative humidity was controlled at 55 ± 5%. The diets were formulated according to the nutrient requirements for laying hens as recommended by the Agricultural Trade Standardization of China (NY/T33-2004), and the composition and nutrient levels of the diets are presented in Table 1. The feed intake was limited to 110 g/day, and all hens had free access to drinking water throughout the day. The experiment lasted for 8 weeks after a 2-week adaptation period. At the end of 28 weeks, one bird was randomly selected from each replicate cage and then killed by jugular exsanguination. Cecal chyme samples were collected aseptically and snap-frozen in liquid nitrogen. The samples were stored at −80°C for analyses including apparent digestibility of nutrients, high-throughput sequencing, short-chain fatty acid (SCFA) profiles and nontargeted metabolomics.

**Table 1:** Composition and nutrient level of the experimental diets.

RB type	HRB^a^		FHRB^b^	
RB level	2.5%	5.0%	2.5%	5.0%
*Ingredient (%)*				
Corn	58.40	56.80	58.40	56.80
Soybean meal	25.20	24.40	25.20	24.40
Limestone	8.00	8.00	8.00	8.00
Soybean oil	0.40	0.30	0.40	0.30
HRB	2.50	5.00	—	—
FHRB	—	—	2.50	5.00
Premix^c^	5.00	5.00	5.00	5.00
TiO_2_	0.50	0.50	0.50	0.50
*Nutrient level*				
Total energy (MJ/kg)	13.02	13.15	12.71	13.02
Metabolic energy (MJ/kg)	12.42	12.42	12.42	12.42
Crude protein (%)	20.42	20.20	20.01	20.22
Ether extract (%)	2.97	3.14	2.97	3.14
Crude fiber (%)	3.20	3.82	3.34	3.43
Ash (%)	17.20	17.39	17.52	17.75
Calcium (%)	4.67	4.79	4.97	5.00
Phosphorus (%)	0.70	0.74	0.76	0.80

a HRB, heat-treated rice bran.

b FHRB, fermented heat-treated rice bran.

c The premix provided the following per kilogram of diets: VA 10,000 IU, VD_3_ 3,000 IU, VE 25 mg, VK_3_ 2.5 mg, VB_2_ 6 mg, VB_6_ 3 mg, VB_12_ 0.01 mg, biotin 0.1 mg, pantothenic acid 10 mg, folic acid 0.3 mg, nicotinamide 40 mg, Cu (as copper sulfate) 15 mg, Fe (as ferrous sulfate) 90 mg, Mn (as manganese sulfate) 110 mg, Zn (as zinc sulfate) 90 mg, I (as potassium iodide) 90 mg, and Se (as selenium yeast) 0.3 mg.

### Laying performance

The numbers and weights (g) of the eggs laid were recorded every day at 8:00 am and 4:00 pm during the experimental period. The feed consumed was recorded once a week. These production data were continuously tracked for 8 weeks to calculate the average egg weight (AEW), egg production (EP), average daily feed intake (ADFI) and feed conversion ratio (FCR, feed intake/egg weight) for the three stages during weeks 21–24, weeks 25–28, and weeks 21–28 of the experimental period.

### Apparent digestibility of nutrients

On the 53rd to 56th days of the experiment, the feces of each cage of laying hens were collected every day, and 10% sulfuric acid was added for nitrogen fixation. The samples were store at −20°C. After the test, all the fecal samples in each cage were mixed evenly, dried at 65°C to constant weight, pulverized, passed through a 40-mesh sieve, and stored at −20°C (Wan et al., 2021). Titanium dioxide (TiO_2_) was then used as an exogenous indicator to calculate the apparent digestibility of nutrients (Duarte et al., 2019).

### DNA extraction, 16S rDNA sequencing, and bioinformatics analysis

The total genomic DNA was extracted from the cecal chyme samples with a QIAamp Fast DNA Stool Mini Kit (QIAGEN, Hilden, Germany) and quantified with a NanoDrop spectrophotometer (Thermo Fisher Scientific Inc., Wilmington, DE, United States). The purity of DNA was detected by 2% agarose gel electrophoresis. The bacterial 16S rDNA V4 region was amplified based on the specific primers 515F (5′-GTGCCAGCMGCCGCGGTAA-3′) and 806R (5′-GGACTACHVGGGTWTCTAAT-3′). The purified PCR amplicons were paired-end sequenced using the Illumina NovaSeq 6000 platform (Illumina Inc., San Diego, CA, United States). Then, using DATA2 method^1^ for sequence denoising. Call qiime cutadapt trim-paired to cut off the primer fragment of the sequence and discard the sequence without matching the primer; call DADA2 through qiime dada2 denoise-paired for quality control, denoising, splicing, and chimerism removal. Then signature sequences of OTUs were merged and the sequencing quantity of each sample was counted (Supplementary Table S2).

Based on the Greengene database (Release 13.8)^2^ and the classify-sklearn algorithm of QIIME2^3^, the representative sequences of each OTU obtained are compared with the database for similarity and species annotation, and the community composition of each sample at different classification levels is calculated. Using the method of Rarefaction, by calling the qiime diversity alpha reconstruction command, the minimum flattening depth is set to 10, and the maximum flattening is set to 95% of the sequence size with the lowest sequencing depth among all samples for OTU table flattening. Then used for alpha diversity and beta diversity analysis and intermediate data was provided in the supplementary file (Supplementary Table S3). All procedures were performed by Suzhou PANOMIX Biomedical Tech Co., Ltd.

### Short-chain fatty acid analysis

Place 0.50 g cecal chyme into a 2 ml centrifuge tube, diluted with 1 ml ultrapure water, homogenized, and then centrifuged at 12,000 rpm for 10 min. The supernatant was added with 25% (w v^−1^) metaphosphoric acid solution, and after centrifugation, the supernatant was aspirated using 0.22 μm filter membrane. Concentrations of SCFA in cecal chyme of laying hens were determined by gas chromatograph (GC-14B, Shimadzu Corporation, Japan; DB-FFAP capillary column: 30.00 m × 0.32 mm × 0.25 μm film thickness).

### Sample preparation and LC–MS analysis

Chyme samples (100 mg) were put into a 2 ml centrifuge tube, 600 μl of methanol (4 ppm) was added, and the tube was vortexed for 30 s. Additionally, 100 mg of glass beads was put into a tissue grinder for 90 s at 60 Hz, and after 10 min of room temperature ultrasound, the supernatant was filtered through a 0.22 μm membrane and put into a detection bottle for LC–MS detection.

The Vanquish UHPLC System was used to conduct the LC analysis (Thermo Fisher Scientific, United States). Chromatography was performed using a 150 mm × 2.1 mm, 1.8 μm ACQUITY UPLC^®^ HSS T3 Column (Waters, Milford, MA, United States). The column was kept at a constant 40°C. The flow rate and injection volume were set at 0.25 ml/min and 2 μl (Zelena et al., 2009). Mass spectrometric detection of metabolites was performed on a Q Exactive Orbitrap (Thermo Fisher Scientific, United States) with an ESI ion source. This was done using simultaneous MS1 and MS/MS capture (Full MS-ddMS2 mode, data-dependent MS/MS). The following parameters were used: automatic dynamic exclusion time; capillary temperature of 325°C; MS1 range of *m*/*z* 81–1,000; MS1 resolving power of 70,000 FWHM; number of data-dependent scans per cycle of 10; and MS/MS resolving power of 17,500 FWHM (Want et al., 2013).

### LC–MS data processing and differential metabolite identification

The raw data were firstly converted to mzXML format by MSConvert in ProteoWizard software package and processed using XCMS for feature detection, retention time correction, and alignment (Smith et al., 2006; Darren et al., 2008). The metabolites were identified by accurate mass spectrometry and MS data, which were matched with HMDB^4^, Massbank^5^, LipidMaps^6^ and KEGG^7^ (Ogata et al., 1999).

All modeling and analyses of multivariate data were performed using Ropls of the R software package. Partial least-square discriminant analysis (PLS-DA) was used to build the models (Boulesteix and Korbinian, 2007). PLS-DA allowed the identification of differential metabolites using the variable importance on projection (VIP) and *p*-value from a two-tailed Student’s *t*-test on the normalised peak areas. Finally, *p* < 0.05 and VIP >1 were considered to be statistically significant metabolites. Differential metabolites were subjected to pathway analysis by MetaboAnalyst 5.0^8^ which combines results from powerful pathway enrichment analysis pipelines.

### Statistical analysis

Test data are expressed as the means ± SD. Two-way ANOVA was performed using GraphPad Prism V 8.0.1 (GraphPad Software, San Diego, California, United States) and SPSS 21.0 statistical software (IBM Corp., Chicago, IL, United States) as a 2 × 2 factorial arrangement (two levels of HRB and two levels of FHRB). Data analysis included the main effects of rice bran type and feeding level, and their interaction. Duncan’s multiple comparison test was used to separate means when the interactive effects significantly different, and *p* < 0.05 was considered a significant difference standard (Wu et al., 2018; Tang et al., 2022). Spearman’s rank correlation coefficient was used to analyze the relationship between the major changes in the intestinal microbiota and apparent digestibility of nutrients and differentially expressed metabolites. These correlation networks were visualized using Cytoscape 3.9.1 software (Smoot et al., 2011).

## Results

### Production performance

FHRB supplementation had no significant effects on egg production (EP), average egg weight (AEW) and feed-to-egg ratio (FCR), but significantly increased the average daily feed intake (ADFI) from 25 to 28 weeks (Table 2). Additionally, 5.0% feeding level increased EP and AEW, and decreased FCR significantly during the experiment (*p* < 0.05). There were no interactions between probiotic fermentation and feeding level on the performance of laying hens.

**Table 2:** Effects of RB type and feeding level on the production performance of laying hens.

RB type	HRB		FHRB		*p*-value		
RB level	2.5%	5.0%	2.5%	5.0%	Probiotic fermentation	Feeding level	Interaction
*EP (%)*							
21 to 24 weeks	52.68 ± 11.49	49.93 ± 11.61	50.34 ± 9.90	50.00 ± 7.93	0.727	0.434	0.314
25 to 28 weeks	82.57 ± 6.81	87.39 ± 3.18	86.16 ± 3.27	90.40 ± 2.60	0.153	0.031	0.611
21 to 28 weeks	67.63 ± 7.00	68.66 ± 5.97	68.25 ± 6.05	70.03 ± 3.87	0.703	0.036	0.300
*AEW (g/hen*…*d)*							
21 to 24 weeks	50.65 ± 4.22	52.93 ± 5.79	51.01 ± 4.24	52.96 ± 5.71	0.477	0.405	0.359
25 to 28 weeks	56.02 ± 1.83	57.43 ± 1.61	56.09 ± 1.51	57.29 ± 1.47	0.386	0.078	0.600
21 to 28 weeks	53.33 ± 1.01	55.18 ± 1.39	53.55 ± 1.29	55.13 ± 1.74	0.078	0.021	0.969
*ADFI (g/hen*…*d)*							
21 to 24 weeks	98.88 ± 0.77	98.34 ± 1.83	98.71 ± 0.87	98.98 ± 0.45	0.928	0.813	0.839
25 to 28 weeks	97.41 ± 2.82	97.99 ± 4.27	98.74 ± 1.63	98.22 ± 0.81	0.039	0.118	0.396
21 to 28 weeks	97.64 ± 1.74	98.15 ± 1.00	98.72 ± 1.70	98.60 ± 0.61	0.598	0.198	0.883
*FCR (g/g)*							
21 to 24 weeks	1.95 ± 0.29	1.86 ± 0.25	1.94 ± 0.38	1.87 ± 0.35	0.177	0.759	0.618
25 to 28 weeks	1.74 ± 0.04	1.71 ± 0.05	1.76 ± 0.06	1.71 ± 0.06	0.900	0.420	0.785
21 to 28 weeks	1.85 ± 0.15	1.78 ± 0.18	1.84 ± 0.20	1.79 ± 0.13	0.206	0.045	0.968

HRB, heat-treated rice bran; FHRB, fermented heat-treated rice bran; EP, egg production; AEW, average egg weight; ADFI, average daily feed intake. All values are expressed as the means with standard (*n* = 8). *p* < 0.05 indicates that the main effect and interaction have significant difference. Based on interaction *p* < 0.05.

### Apparent digestibility of nutrients

The nutritional apparent digestibility in laying hens was shown in Table 3. During the experimental period, feeding FHRB significantly increased the apparent digestibility of dry matter (DM), crude protein (CP), ether extract (EE) and crude fiber (CF) of laying hens (*p <* 0.05), while these indices were not significantly affected by feeding level (*p* > 0.05). Moreover, there was an interaction in the ash apparent digestibility (*p <* 0.05), which was significantly higher in the 5.0% FHRB group.

**Table 3:** Effects of RB type and feeding level on apparent digestibility of nutrients in laying hens.

RB type	HRB		FHRB		*p*-value		
RB level	2.5%	5.0%	2.5%	5.0%	Probiotic fermentation	Feeding level	Interaction
GE	77.61 ± 4.88	74.28 ± 3.91	76.01 ± 3.41	74.94 ± 5.88	0.776	0.189	0.494
DM	68.46 ± 6.21	74.14 ± 3.13	76.61 ± 5.23	74.90 ± 5.63	0.022	0.288	0.053
CP	52.76 ± 5.54	55.79 ± 4.31	57.40 ± 5.04	59.69 ± 7.78	0.047	0.207	0.859
EE	64.05 ± 2.64	65.38 ± 3.50	69.79 ± 2.98	68.62 ± 2.86	<0.001	0.943	0.251
CF	30.95 ± 4.58	33.81 ± 2.85	36.92 ± 3.45	38.04 ± 4.6	0.001	0.165	0.535
Ash	41.23 ± 3.81^ab^	37.85 ± 3.14^b^	40.29 ± 2.73^ab^	43.49 ± 5.21^a^	0.095	0.946	0.022
Ca	54.02 ± 4.69	57.91 ± 3.28	57.78 ± 5.77	57.26 ± 3.35	0.327	0.288	0.167
TP	34.53 ± 5.56	38.33 ± 3.36	36.75 ± 4.51	39.36 ± 6.78	0.384	0.093	0.749

HRB, heat-treated rice bran; FHRB, fermented heat-treated rice bran; GE, gross energy; DM, dry matter; CP, crude protein; EE, ether extract; CF, crude fiber; Ca, calcium; TP, total phosphorus. All values are expressed as the means with standard (*n* = 8). *p* < 0.05 indicates that the main effect and interaction have significant difference. Based on interaction *p* < 0.05, ^a, b^ values with different letters are significantly different at *p* < 0.05.

### Cecal microbiota

To investigate the effect of probiotic fermentation on the composition of the cecal microbiota, fresh chyme samples from 32 laying hens were collected to amplify and sequence. A total of 1,811,022 high-quality sequences were classified as bacteria with an average length of 315 bp. And raw sequence data generated, quality filtration, chimera filtration and final clean merged reads used in the analysis were shown in supplementary file (Supplementary Table S2).

**Figure 1:**
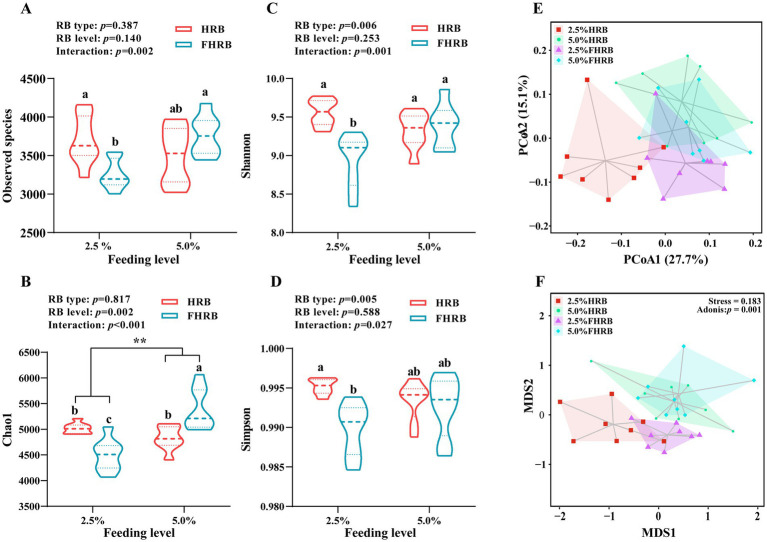
Effects of RB type and feeding level on the cecal microbial diversity in laying hens. (**A–D**, alpha diversity; **E,F**, beta diversity). **(A)** Observed species. **(B)** Chao1. **(C)** Shannon. **(D)** Simpson. **(E)** Principal coordinate analysis (PCoA). **(F)** NMDS analysis. All values are expressed as the means with standard (*n* = 8). *p* < 0.05 indicates that the main effect and interaction have significant differences. * and ** indicate significant (*p* < 0.05) and highly significant (*p* < 0.01) differences at feeding level, respectively. Based on interaction *p* < 0.05, ^a–c^ values with different letters are significantly different at *p* < 0.05.

Firstly, we analyzed the effects of FHRB and feeding level on the microbial diversity of cecal chyme (Figure 1). The observed species in the 2.5% FHRB group were significantly lower than those in the 2.5% HRB and 5.0% FHRB groups (Figure 1A). Dietary supplemented with 5.0% of HRB and FHEB increased the Chao1 index compared to 2.5% feeding level, and multiple comparisons also revealed that the 5.0% FHRB group had a higher Chao1 index than other three groups (Figure 1B, *p* < 0.05). Compared with the HRB group, the FHRB group had significantly lower Shannon and Simpson indices (Figures 1C,D). Additionally, probiotic fermentation and feeding level had interactive effects on the Shannon and Simpson indices. Multiple comparisons revealed that the Shannon index of the 2.5% FHRB group was lower than other three groups, and the Simpson index of the 2.5% FHRB group was lower than 2.5% HRB group. Beta diversity analyses are shown in the PCoA (Figure 1E) and NMDS analysis (Figure 1F), with noticeable changes in the cecal microbiota owing to FHRB and feeding level. The NMDS ordination plot showed that the cecal microbiota in the samples were clearly separated (ANOSIM, *p* = 0.001).

**Figure 2:**
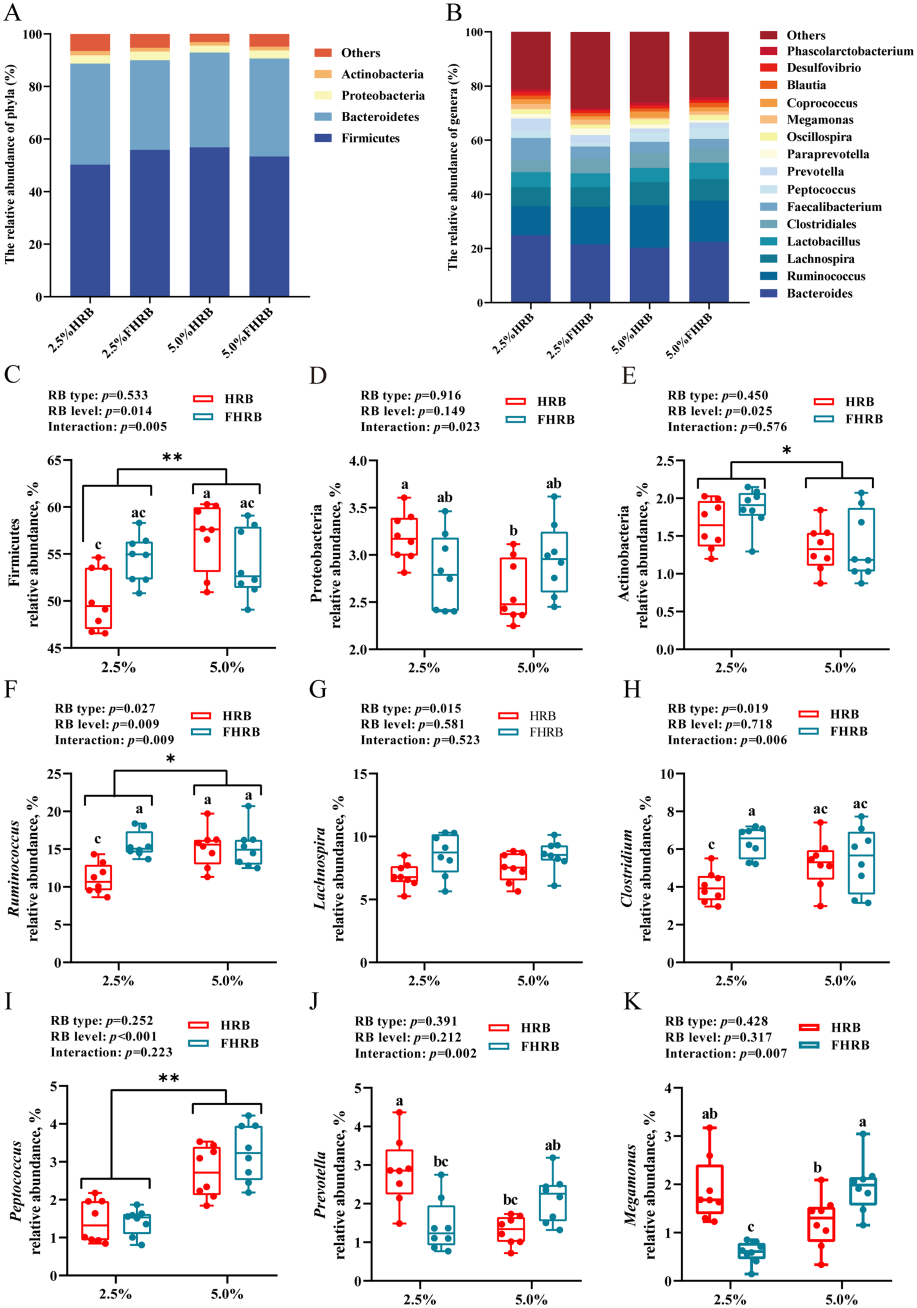
Effects of RB type and feeding level on the cecal microbiota of laying hens. **(A)** The most abundant (relative abundance >1%) phyla of cecal microbiota. **(B)** The most abundant (relative abundance >1%) genera of cecal microbiota. **(C–K)** The relative abundance of Firmicutes, Proteobacteria, Actinobacteria, *Ruminococcus*, *Lachnospira*, *Clostridium*, *Peptococcus*, *Prevotella* and *Megamonas*. * and ** indicate significant (*p* < 0.05) and highly significant (*p* < 0.01) differences at feeding level, respectively. Based on interaction *p* < 0.05, a–c values with different letters are significantly different at *p* < 0.05.

To further determine the effects of FHRB and feeding level on cecal microbiota of laying hens, bacterial taxa with relative abundances >1% were analyzed. At the phylum level (Figure 2A), cecal microbiota were dominated by Firmicutes (49.11%–66.54%), Bacteroidetes (26.17%–39.03%), Proteobacteria (2.03%–5.07%) and Actinobacteria (1.49%–3.37%), with an overall relative abundance of over 80%. At the genus level (Figure 2B), the dominant genera in the cecal chyme were *Bacteroides* (15.42%–30.80%), *Ruminococcus* (8.54%–22.70%), *Lachnospira* (5.65%–12.14%), *Lactobacillus* (2.51%–13.60%) and *Clostridiales* (2.96%–7.73%). The phylum-level analysis revealed that supplying FHRB had no significant effect on the microbiota of cecal chyme, while the diets supplemented with 5.0% HRB and 5.0% FHRB significantly increased the relative abundance of Firmicutes, the relative abundance of which in 5.0% HRB group was significantly higher than that in 2.5% HRB group (Figure 2D, *p* < 0.05). And conversely, the relative abundance of Proteobacteria was significantly lower (Figure 2D, *p* < 0.05). In addition, the feeding level with 5.0% decreased that of Actinobacteria (Figure 2E, *p* < 0.05). The genus-level analysis of the predominant abundant genera showed that supplementation with FHRB significantly increased the relative abundances of Lachnospira, Ruminococcus and Clostridiales (Figures 2F-H, *p* < 0.05); and feeding 5.0% HRB and 5.0% FHRB increased the relative abundances of Ruminococcus and Peptococcus (Figures 2F,I, *p* < 0.05). Dietary probiotic fermentation and feeding level interacted with the relative abundances of *Ruminococcus*, *Clostridiales*, *Prevotella* and *Megamonas*. Multiple comparisons showed that the relative abundances of *Ruminococcus* and *Clostridiales* were the lowest in the 2.5% HRB group, and the relative abundances of *Prevotella* and *Megamonas* in 2.5% HRB and 5.0% FHRB groups were higher than those in the other two groups (Figures 2J,K, *p* < 0.01).

### Cecal SCFA concentrations

To evaluate whether alterations in cecal microbiota impacted fermentability, the concentrations of SCFAs in cecal chyme were determined (Table 4). Dietary supplementation with FHRB significantly increased the concentrations of total SCFAs, acetate and valerate (*p <* 0.05). Compared with the 2.5% level group, feeding 5.0% of HRB and FHRB significantly increased the concentrations of all SCFAs except acetate (*p <* 0.05). In addition, there was an interaction between probiotic fermentation and feeding level in the concentration of total SCFAs, acetate and propionate (*p* < 0.01). The concentrations of total SCFA and acetate in the 5.0% FHRB group were significantly higher than those in the other three groups (*p* < 0.05), and the concentration of propionate in the 5.0% FHRB group was significantly higher than that in 5.0% HRB group (*p* < 0.05).

**Table 4:** Effects of RB type and feeding level on short-chain fatty acids in cecal chyme of laying hens.

RB type	HRB		FHRB		*p*-value		
RB level	2.5%	5.0%	2.5%	5.0%	Probiotic fermentation	Feeding level	Interaction
Total SCFAs	67.74 ± 8.86^b^	58.45 ± 7.11^c^	59.88 ± 6.13^c^	84.07 ± 6.35^a^	0.007	0.002	<0.001
Acetate	47.78 ± 6.16^b^	40.80 ± 5.95^c^	37.78 ± 3.18^c^	58.47 ± 5.54^a^	0.001	0.052	<0.001
Propionate	12.76 ± 2.56^ab^	10.45 ± 2.03^b^	12.73 ± 2.79^ab^	15.50 ± 2.69^a^	0.797	0.009	0.009
Butyrate	4.48 ± 0.84	3.65 ± 0.90	4.97 ± 0.79	5.20 ± 1.20	0.390	0.005	0.128
Valerate	0.98 ± 0.22	1.40 ± 0.68	1.19 ± 0.42	1.92 ± 0.45	0.002	0.040	0.371
Isobutyrate	0.81 ± 0.16	0.81 ± 0.25	1.41 ± 1.02	1.28 ± 0.23	0.742	0.010	0.737
Isovalerate	0.92 ± 0.19	1.29 ± 0.52	1.79 ± 0.52	1.68 ± 0.48	0.417	0.001	0.144

HRB, heat-treated rice bran; FHRB, fermented heat-treated rice bran; SCFAs, short-chain fatty acids. All values are expressed as the means with standard (*n* = 8). *p* < 0.05 indicates that the main effect and interaction have significant difference. Based on interaction *p* < 0.05, ^a–c^ values with different letters are significantly different at *p* < 0.05.

### Cecal metabolite profiles

**Figure 3:**
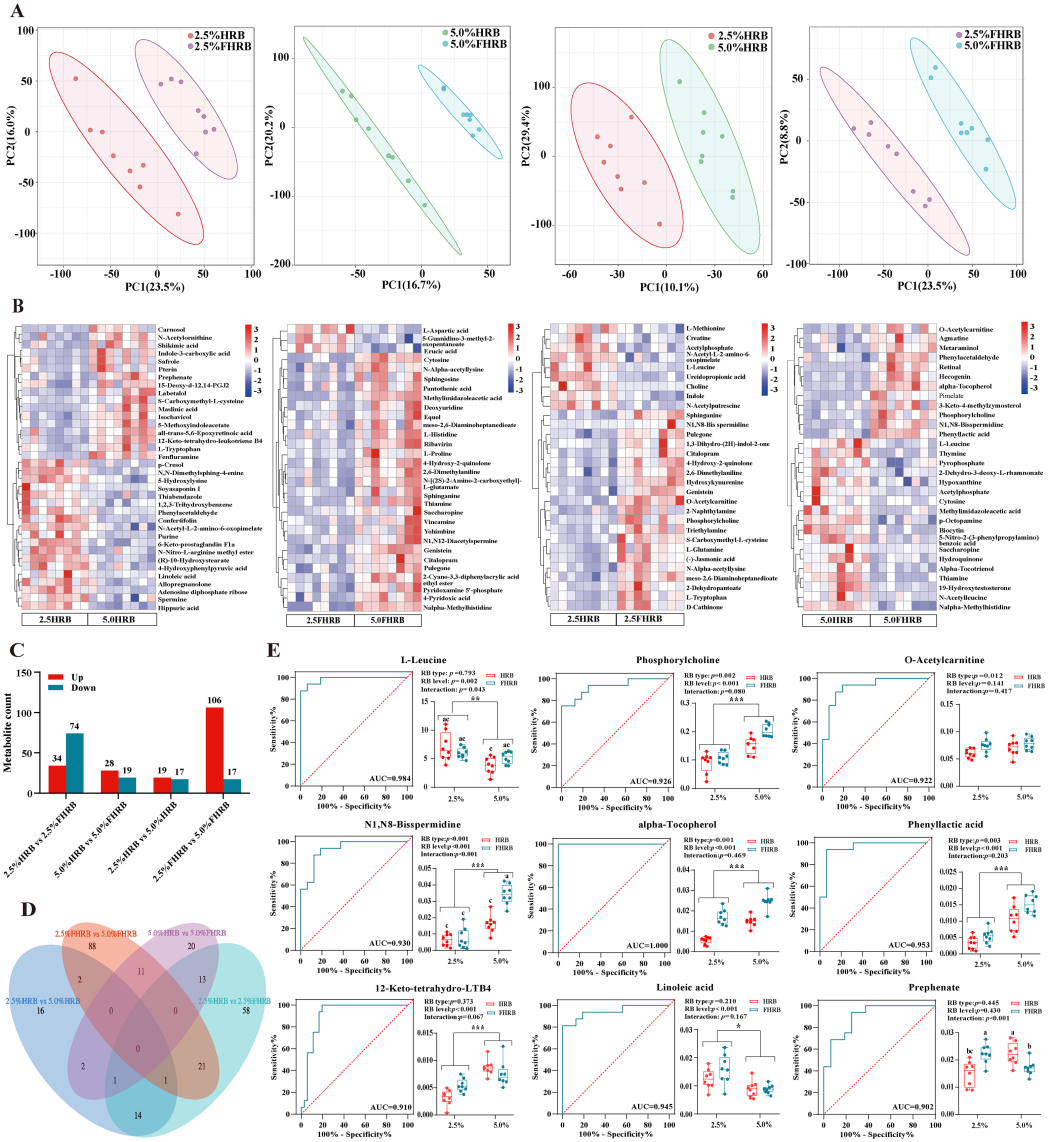
Effects of RB type and feeding level on the cecal metabolic profiles of laying hens. **(A)** Partial least squares projection to latent structures and discriminant analysis (PLS-DA) based on the LC-MS metabolite profiles of the cecal compound data. **(B)** Differential metabolite clustering heatmap of cecal chyme in the two groups (top 30). **(C)** Statistics of differentially expressed metabolites. **(D)** Venn diagram of differential metabolites between different groups. **(E)** ROC analysis of nine different biomarker metabolites based on AUC values ≥90% as potential biomarkers for the effects of RB type and feeding levels in laying hens. * and * * indicate significant (*P* < 0.05) and highly significant (*P* < 0.01) differences at feeding level, respectively. Based on interaction *p* < 0.05, a–c values with different letters are significantly different at *p* < 0.05. *, ** and *** indicate significant (*p* < 0.05), highly significant (*p* < 0.01) and extremely significant (*p* < 0.001) differences at feeding level, respectively. Based on interaction *p* < 0.05, a–c values with different letters are significantly different at *p* < 0.05.

Nontargeted metabolomics analysis identified a total of 5,087 metabolites in the cecal chyme. PLS-DA score plots displayed markedly separated clusters between the two comparison groups, indicating that metabolite profiles of cecal chyme were considerably changed due to FHRB and feeding level (Figure 3A). Taking VIP >1 and *p* < 0.05 as the screening criteria, a total of 314 differential metabolites were identified (Supplementary Table S4). The clustering heatmap showed the differential metabolites with relative abundance in the top 30 of the two groups (Figure 3B). Number of differentially expressed metabolites identified between the groups in Figure 3C. Specifically, a total of 108 differential metabolites were identified in the 2.5% HRB and 2.5% FHRB groups (34 upregulated, 74 downregulated), and 47 differential metabolites were identified between the 5.0% HRB and 5.0% FHRB groups (28 upregulated, 19 downregulated). Thirty-six metabolites were significantly altered between the 2.5 and 5.0% HRB groups (19 upregulated, 17 downregulated), and 123 metabolites were identified between the 2.5 and 5.0% FHRB groups (106 upregulated, 17 downregulated). Among these metabolites, combining Venn diagram and ROC analysis with AUC ≥90% (Figures 3D,E), we identified six coexpressed differential metabolites, including L-leucine, phosphorylcholine, O-acetylcarnitine, N1, N8-bisspermidine, alpha-tocopherol, and phenyllactic acid, as biomarkers for the effects of rice bran type on laying hens (2.5% HRB versus 2.5% FHRB and 5.0% HRB versus 5.0% FHRB). In addition to L-leucine, feeding FHRB significantly increased the levels of other metabolites. In addition, 12-keto-tetrahydro-leukotriene B4, linoleic acid, and prephenate, three coexpressed differential metabolites, were evaluated as biomarkers for the impact of rice bran feeding levels on laying hens (2.5% HRB versus 5.0% HRB and 2.5% FHRB versus 5.0% FHRB). Compared with the 2.5% feeding level, the 5.0%RB group had higher 12-Keto-tetrahydro-leukotriene B4 and lower linoleic acid.

### Enrichment analysis of differentially expressed metabolites in cecal chyme

**Figure 4:**
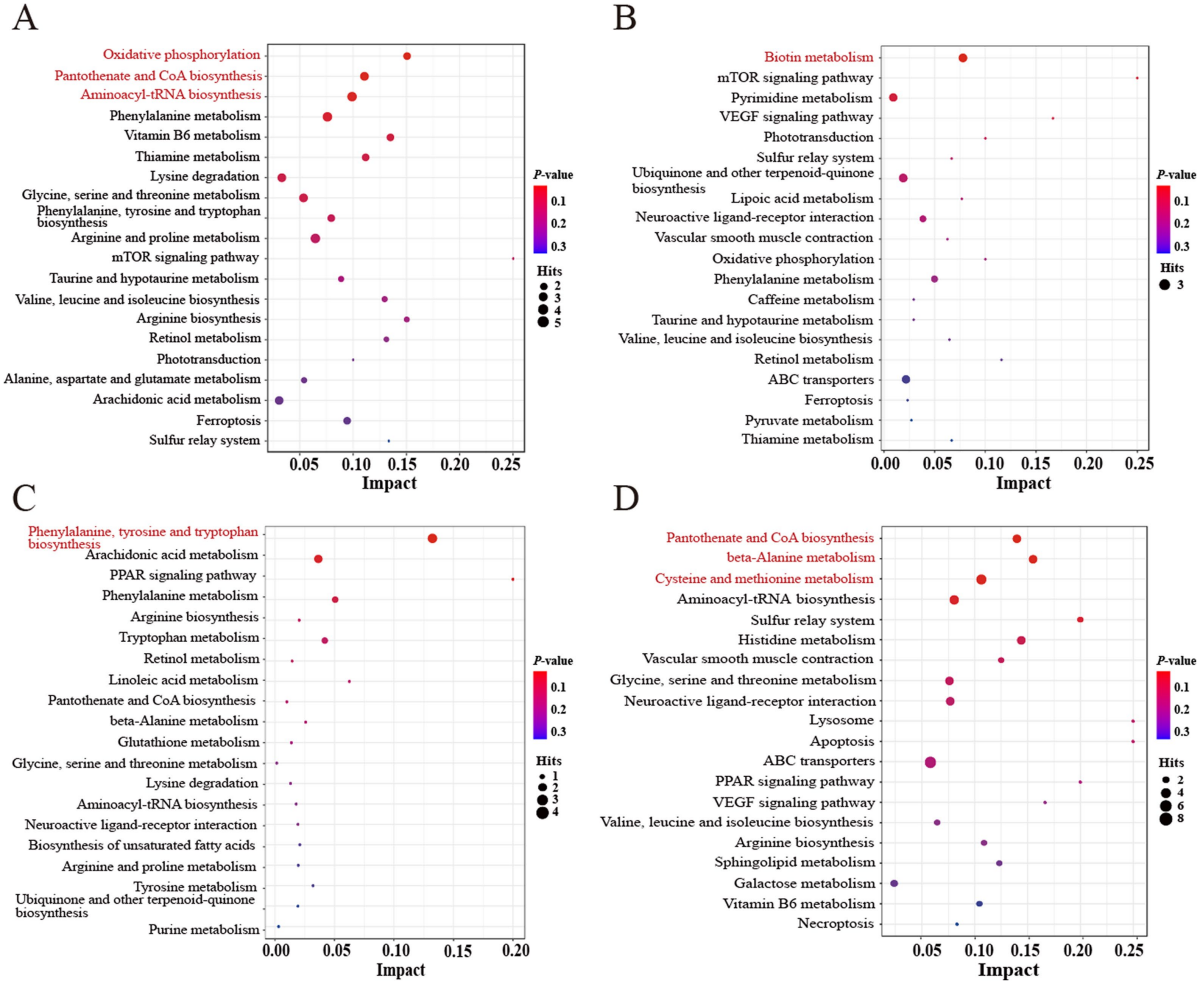
Enrichment analysis of differential metabolites in cecal chyme of laying hens. Significantly changed pathways based on enrichment and topology analysis are shown. The *X*-axis represents pathway impact, and the *Y*-axis represents pathway enrichment. Larger sizes and darker colors represent higher pathway impact values and higher pathway enrichment, respectively. The red pathway indicates the significantly different metabolic pathways between the two groups. **(A)** Metabolic pathways between 2.5% HRB and 2.5% FHRB groups. **(B)** Metabolic pathways between the 5.0% HRB and 5.0% FHRB groups. **(C)** Metabolic pathways between 2.5% and 5.0% HRB groups. **(D)** Metabolic pathways between 2.5% and 5.0% FHRB groups.

To explore the different metabolic pathways that respond to FHRB and feeding level, we imported the significantly different metabolites into KEGG for enrichment analysis. As shown in the metabolome view map (Figure 4), the relevant metabolic pathways were mainly involved in amino acid metabolism, energy metabolism, fatty acid metabolism and other related pathways (Supplementary Table S5). The differential metabolites in the 2.5% HRB and 2.5% FHRB groups were involved in 49 different metabolic pathways in total (Figure 4A), with oxidative phosphorylation, pantothenate and CoA biosynthesis, and aminoacyl-tRNA biosynthesis being considerably enriched. A total of 34 metabolic pathways were identified between the 5.0% HRB and 5.0% FHRB groups (Figure 4B), and the significantly enriched metabolic pathway was biotin metabolism. Moreover, at different RB feeding levels, the coexpressed differential metabolites in the HRB and FHRB groups were mainly enriched in valine, leucine and isoleucine biosynthesis and degradation, ABC transporters, mTOR signaling pathway, aminoacyl-tRNA biosynthesis, ubiquinone, and other terpenoid-quinone biosynthesis. A total of 21 metabolic pathways were involved in differential metabolites between the 2.5% and 5.0% HRB groups (Figure 4C), with the significantly enriched metabolic pathway being phenylalanine, tyrosine and tryptophan biosynthesis. Fifty-six metabolic pathways were engaged in the differential metabolites found in the 2.5% and 5.0% FHRB groups (Figure 4D), with pantothenate and CoA biosynthesis, β-alanine metabolism, cysteine and methionine metabolism being the significantly enriched pathways. Furthermore, the coexpressed differential metabolites between the 2.5% and 5.0% RB groups were mainly enriched in phenylalanine, tyrosine, and tryptophan biosynthesis, arachidonic acid metabolism, PPAR signaling pathway, linoleic acid metabolism and unsaturated fatty acid biosynthesis.

### Correlations between cecal microbiota, metabolites and apparent digestibility of nutrients

**Figure 5:**
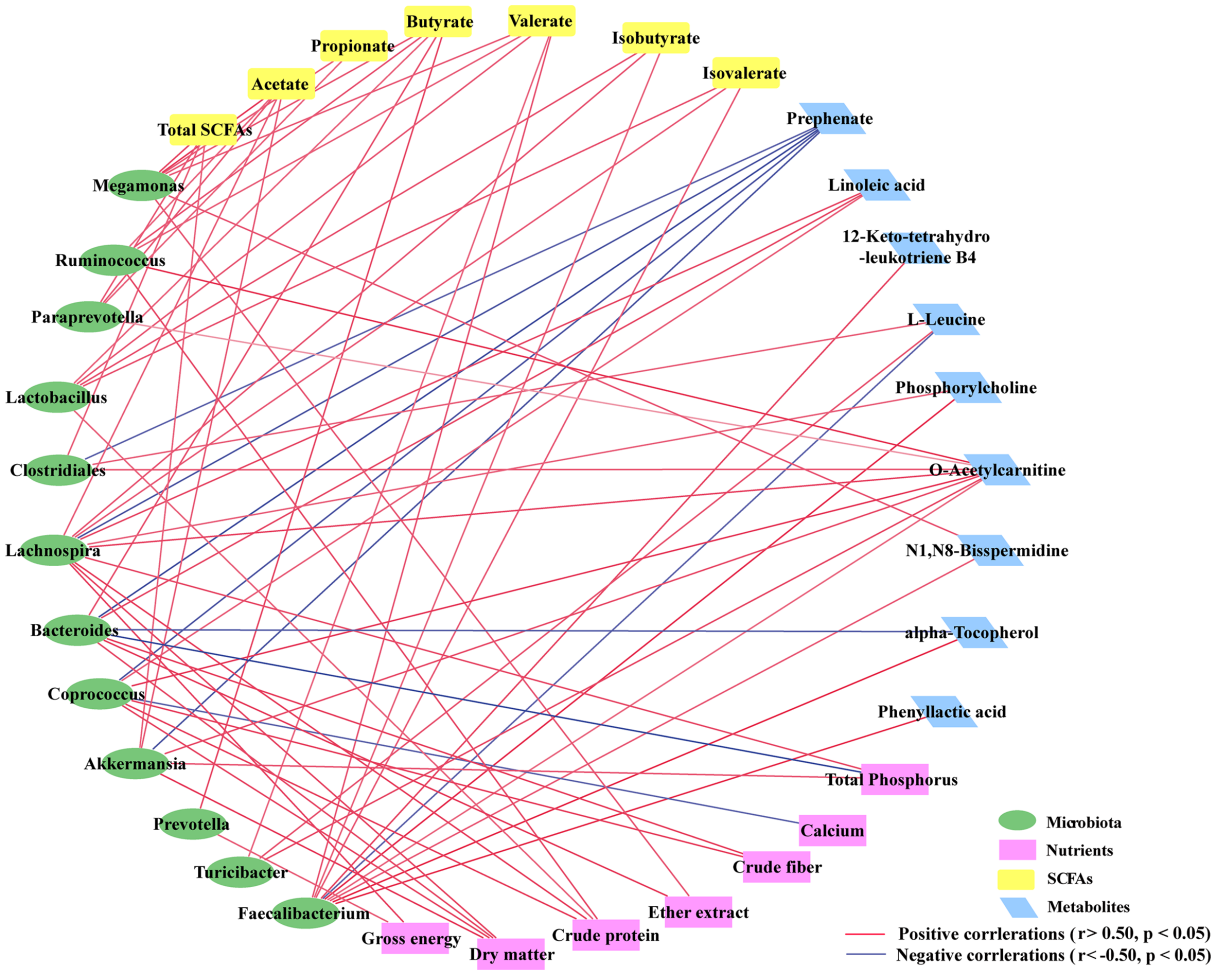
Correlation network analysis among the cecal microbiota (at the genus level), differentially expressed metabolites and apparent digestibility of nutrients. Each line has an absolute Spearman rank correlation; red lines represent positive correlations (*r* > 0.50, *p* < 0.05), and blue lines represent negative correlations (*r* < −0.50, *p* < 0.05).

To investigate how the cecal microbiota is related to different patterns of metabolites and apparent digestibility of nutrients due to alterations in FHRB and feeding level, we used Spearman rank correlation coefficients to analyze the correlations among cecal predominant bacteria at the genus level, metabolites and apparent digestibility of nutrients (Figure 5). Except for *Clostridiales*, *Lachnospira*, *Bacteroides*, *Coprococcus*, and *Akkermansia*, which were negatively correlated with prephenate, *Faecalibacterium* was negatively correlated with L-leucine, and *Bacteroides* was negatively correlated with alpha-tocopherol. The relative abundances of microbiota were positively correlated with SCFAs and differential metabolites. Furthermore, with the exception of calcium and total phosphorus, there were positive correlations between the cecal microbiota and apparent digestibility of nutrients. Collectively, these results indicated that the changes in the cecal microbiota were correlated with alterations of metabolites in laying hens.

## Discussion

China is the largest producer and consumer of rice in the world, producing at least 10 million tons of rice bran annually (Wang et al., 2020). Rice bran, as a major byproduct produced during rice processing, also attracted attention for its potential to provide valuable bioproducts (Punia et al., 2020). To the best of our knowledge, fermentation increased the active compounds of rice bran, and mice orally administrated fermented rice bran altered cecal microbiota (Shibayama et al., 2018). However, the effect of fermented rice bran on the performance and microbial composition of laying hens is still unknown. Therefore, we combined 16S rDNA sequencing and nontargeted metabolomics technology to investigate the effects of rice bran type and feeding level on the possible roles of cecal microbiota and metabolites in laying hens.

Rice bran was used as an alternative feedstuff in laying hen diets, and its feeding level had a significant impact on the egg performance of laying hens, but the recommended addition did not exceed 7.5% (Nobakht, 2007). Therefore, we established two feeding levels of 2.5 and 5.0% to compare the effects of probiotic fermentation on nutritional utilization and egg performance of laying hens. The egg production increased rapidly with the extension of rearing, and feeding FHRB significantly increased the ADFI of laying hens from 25 to 28 weeks. Compared with the 2.5% feeding level group, dietary supplementation with 5.0% HRB and 5.0% FHRB significantly increased EP and AEW, and decreased FCR in the experimental period. Moreover, our results also indicated that feeding FHRB significantly improved the apparent digestibility of DM, CP, EE, and CF in laying hens. This may be because the metabolites produced by *B. subtilis* during rice bran fermentation degrade macromolecules in feed materials into small molecules that can be easily digested and absorbed and *L. plantarum* produced large amount of organic acids, such as lactic acid and acetic acid, which can reduce pH and inhibit the colonization of harmful bacteria and maintain the balance of cecal microbiota, and then have a positive impact on the production performance of laying hens (Kiers et al., 2000; Guo et al., 2021).

Fermented rice bran significantly regulated intestinal microbial composition and amino acid and lipid metabolism to promote nutrient availability and intestinal health in healthy mice (Nealon et al., 2019). In the present study, dietary FHRB supplementation significantly changed the cecal microbiota of laying hens. Specifically, supplementation with FHRB decreased the Shannon and Simpson indices. Shannon and Simpson indices reflect the flora diversity, and supplementation with FHRB decreased the above indices possibly because the reproduction of cecal *Lachnospiraceae*, *Clostridium* and other probiotics inhibited the production of hybrids. The analysis of beta diversity also showed that the cecal microbiota in the samples were clearly separated. Therefore, we further analyzed the cecal microbiota. At the phylum level, similar to previous findings, Firmicutes and Bacteroidetes were the absolute dominant phyla, followed by Proteobacteria and Actinobacteria. In this study, feeding FHRB had no significant difference in microbial composition, while dietary supplementation with 5.0% HRB and 5.0%FHRB significantly increased the relative abundance of Firmicutes. Firmicutes is closely related to the energy utilization and fat synthesis of the host, and changes in the relative abundance of Firmicutes to Bacteroidetes may promote the host to extract dietary energy from the feed and promote lipogenesis (Turnbaugh et al., 2006; He et al., 2019) which may be related to the fat acid profile and fermented probiotics in the rice bran. At the genus level, the dominant genera in the cecal chyme of laying hens were *Bacteroides*, *Ruminococcus*, *Lachnospira*, *Lactobacillus* and *Clostridiales*. Similar to previous studies, the relative abundances of *Lachnospira* and *Clostridiales* were significantly increased by adding FHRB to the diet (Nealon et al., 2019). Dietary supplementation with 5.0% level significantly increased the relative abundances of *Ruminococcus* and *Peptococcus*. It was well known that microbiota such as *Lachnospira*, *Clostridium*, *Ruminococcus*, and *Peptostreptococcus* are crucial producers of SCFAs (Koh et al., 2016; Shang et al., 2018; He et al., 2019), and their higher relative abundances are helpful to maintain intestinal homeostasis, enhance intestinal absorption and utilization of nutrients, and prevent intestinal inflammation (He et al., 2020; Zou et al., 2020). The SCFA data revealed that feeding FHRB increased the concentrations of acetate, valerate and total SCFAs, while feeding 5.0% of HRB and FHRB resulted in higher concentrations of all SCFAs except acetate. Short-chain fatty acids are produced by microbial fermentation of dietary fiber, which is closely related to host energy metabolism (den Besten et al., 2013). We speculated that a diet supplemented with a relatively higher level of FHRB is conducive to promoting the production of intestinal SCFAs and maintaining a host-friendly gut environment.

Given the significant differences in cecal microbiota, we next investigated the effects of rice bran type and feeding level on cecal metabolites. Metabolites reflect the final state of animal digestion, absorption and metabolism of dietary nutrients (Zhang et al., 2019). Dietary fiber, protein and peptides that have not been digested in the small intestine are further fermented and metabolized by microbiota in the cecum (Fan et al., 2015). On the basis of ROC analysis with AUC ≥90%, six coexpressed differential metabolites were identified as biomarkers for the probiotic properties of FHRB in laying hens, including L-leucine, phosphorylcholine, acetylcarnitine, O-acetylcarnitine, N1, N8-bisspermidine, and alpha-tocopherol. Glutamine is an amino acid with a variety of important metabolic functions, enhances body immune function and antioxidant capacity, and is an energy source in the gastrointestinal tract (Cruzat et al., 2018). L-leucine is a branched-chain amino acid that synthesizes glutamine, and its reduced content indicates that feeding fermented rice bran can accelerate branched chain fatty acid breakdown in laying hens, possibly related to body energy supply. Phosphatidylcholine is a specific endogenous ligand of PPARα, which regulates the expression of lipid metabolism genes, participates in daytime hepatic uptake of fatty acids and β-oxidation in muscle, and improves the utilization of fatty acids in muscle cells (Liu et al., 2013). In addition, acetylcarnitine is the esterified product of carnitine, together with diamine generated by the hydrolysis of L-arginine by arginine decarboxylase, which serves as a carrier to transport fatty acids to mitochondria for β-oxidation, producing ATP to provide energy, participating in fat metabolic pathways and glucose homeostasis regulation, and increasing the capacity of the antioxidant defense system of the liver (Scafidi et al., 2010; Cahova et al., 2015; Ferreira and Mckenna, 2017). A potent antioxidant, alpha-tocopherol, is considered to be the dominant form of vitamin E, and binds to α-tocopherol transfer protein to prevent its degradation (Peh et al., 2016). Compared with the HRB group, dietary supplementation with FHRB significantly increased the content of alpha-tocopherols, further demonstrating the potential antioxidant function of FHRB in laying hens. The results of the enrichment analysis also indicated that the above metabolites were mainly involved in amino acid and energy metabolism-related pathways, glycerophospholipid metabolism, ubiquinone and other terpene quinone biosynthesis metabolism, and played important roles in body protein breakdown, energy metabolism and physical health. Moreover, by comparing the differential metabolites of 2.5% and 5.0% HRB groups with those of the 2.5% and 5.0% FHRB groups, it was found that the coexpressed metabolites including 12-keto-tetrahydro-leukotriene B4, linoleic acid, and prephenate. Among them, 12-keto-tetrahydro-leukotriene B4 is a lipid mediator derived from the metabolism of arachidonic acid by 5-lipoxygenase, which plays an important role in the regulation of inflammatory disease (Le Bel et al., 2014). Linoleic acid, an essential fatty acid, is an important part of cell membrane structure and function and needs to be obtained from the diet. Supplementation with 5.0% level of HRB and FHRB downregulated linoleic acid, and we speculate that linoleic acid may be utilized by laying hens and is related to improving egg production performance (Wang et al., 2015). These metabolites were mainly enriched in phenylalanine, tyrosine and tryptophan biosynthesis, and fatty acid metabolism-related pathways. We suggest that the feeding level of rice bran may be involved in organismal metabolism by regulating amino acid metabolism and lipid metabolism. Dietary supplementation with FHRB and 5.0% level of supplementation may have potential health-promoting properties for the organism.

The gut microbiota acts in a coordinated manner to achieve metabolic communication with the host (Nicholson et al., 2012), and we continued to explore the correlations between differential microbial genera, biomarker differential metabolites, SCFAs, and apparent digestibility of nutrients. Notably, except for prephenate, the relative abundances of most microbiota were positively correlated with the concentrations of differential metabolites and SCFAs and the apparent digestibility of nutrients. As SCFAs are metabolites of intestinal microbes, we speculated that, in addition to the individual differences between laying hens, the promoting effect of fermented rice bran on the apparent digestibility of nutrients in laying hens may be related to the intestinal SCFA-producing flora, accompanied by higher concentrations of intestinal SCFAs, lower intestinal pH, and the more acidic environment is conducive to the growth of probiotics and the promotion of nutrient digestion and absorption.

## Conclusion

In summary, our study demonstrated that dietary supplementation with FHRB could improve the production performance and apparent digestibility of nutrients in laying hens, with better results at the 5.0% supplementation level. Meanwhile, dietary FHRB supplementation altered the cecal microbiota of laying hens, especially the microbiota associated with the production of SCFAs. These bacteria subsequently changed the concentrations of SCFAs and might be involved in regulating body metabolism through amino acid, energy, and lipid metabolism pathways, which may further improve the apparent digestibility of nutrients and production performance of laying hens.

## Data availability statement

The datasets presented in this study can be found in online repositories. The names of the repository/repositories and accession number(s) can be found at: https://www.ncbi.nlm.nih.gov/, PRJNA922673.

## Ethics statement

The animal study was reviewed and approved by the Animal Care and Use Committee of Nanjing Agricultural University, in compliance with the Chinese guidelines for animal welfare and experimental protocol.

## Author contributions

WY and WJZ conceived this study and contributed to the funding acquisition. YMW participated in the design of the study, analyzed experimental results, and wrote the manuscript. YMW, WD, HF, HH, and HZ performed the animal experiments and collected the data. All authors contributed to the article and approved the submitted version.

## Funding

This study was supported by Jiangsu Key R&D Project in Agriculture (BE2020378) and Jiangsu Agriculture Science and Technology Innovation Fund [CX(19)1006].

## Conflict of interest

The authors declare that the research was conducted in the absence of any commercial or financial relationships that could be construed as a potential conflict of interest.

## Publisher’s note

All claims expressed in this article are solely those of the authors and do not necessarily represent those of their affiliated organizations, or those of the publisher, the editors and the reviewers. Any product that may be evaluated in this article, or claim that may be made by its manufacturer, is not guaranteed or endorsed by the publisher.

## Footnotes

^1^ https://benjjneb.github.io/dada2/tutorial.html

^2^ http://greengenes.secondgenome.com/

^3^ https://github.com/QIIME2/q2-feature-classifier/

^4^ http://www.hmdb.ca

^5^ http://www.massbank.jp

^6^ http://www.lipidmaps.org

^7^ http://www.lipidmaps.org

^8^ http://www.metaboanalyst.ca
