# Supplementary material for: Effect of fermented heat-treated rice bran on performance and possible role of intestinal microbiota in laying hens
Source: Front Microbiol. 2023 Apr 27;14:1144567. doi: 10.3389/fmicb.2023.1144567 (PMC10172586; doi:10.3389/fmicb.2023.1144567)
Supplement: Supplementary file 1 [file Table_1.DOCX]

Supplementary Material

**Effect of fermented heat-treated rice bran on performance and possible role of intestinal microbiota in laying hens**

**Yamei Wang^1^**^†^**, Weijiang Zheng^1^**^†^**, Wei Deng^1^, Hua Fang^1^, Heng Hu^1^, He Zhu^1^, Wen Yao^1,2*^**

*** Correspondence:** Corresponding Author: yaowen67jp@njau.edu.cn

# Supplementary Tables

Table S1 Analysis of L^18^（3^5^）orthogonal test results of fermented rice bran

| Factors / levels /No. | Inoculation quantity (‰)-A | *B. subtilis*:  *L. plantarum-B* | Solid-liquid ratio (m: v)-C | Fermentation temperature (℃)-D | Fermentation time (h)-E | DPPH free radical scavenging activity (%) |
| --- | --- | --- | --- | --- | --- | --- |
| 1 | 0.4 | 2: 8 | 1: 0.5 | 30 | 24 |  |
| 2 | 0.6 | 3: 7 | 1: 0.6 | 35 | 36 |  |
| 3 | 0.8 | 4: 6 | 1: 0.7 | 37 | 48 |  |
| 1 | 0.4 | 2: 8 | 1: 0.5 | 30 | 24 | 85.54±2.11 |
| 2 | 0.4 | 2: 8 | 1: 0.6 | 37 | 36 | 86.75±0.13 |
| 3 | 0.4 | 3: 7 | 1: 0.5 | 35 | 36 | 82.08±1.62 |
| 4 | 0.4 | 3: 7 | 1: 0.7 | 30 | 48 | 89.11±2.79 |
| 5 | 0.4 | 4: 6 | 1: 0.6 | 35 | 24 | 91.43±1.63 |
| 6 | 0.4 | 4: 6 | 1: 0.7 | 37 | 48 | 78.19±0.15 |
| 7 | 0.6 | 2: 8 | 1: 0.5 | 35 | 48 | 83.25±1.23 |
| 8 | 0.6 | 2: 8 | 1: 0.7 | 30 | 24 | 89.19±2.57 |
| 9 | 0.6 | 3: 7 | 1: 0.6 | 37 | 24 | 78.60±2.33 |
| 10 | 0.6 | 3: 7 | 1: 0.7 | 35 | 36 | 86.47±1.65 |
| 11 | 0.6 | 4: 6 | 1: 0.5 | 37 | 48 | 81.41±0.95 |
| 12 | 0.6 | 4: 6 | 1: 0.6 | 30 | 36 | 81.59±3.28 |
| 13 | 0.8 | 2: 8 | 1: 0.6 | 35 | 48 | 82.75±0.53 |
| 14 | 0.8 | 2: 8 | 1: 0.7 | 37 | 36 | 86.78±2.82 |
| 15 | 0.8 | 3: 7 | 1: 0.5 | 37 | 24 | 90.65±2.50 |
| 16 | 0.8 | 3: 7 | 1: 0.6 | 30 | 48 | 82.50±0.44 |
| 17 | 0.8 | 4: 6 | 1: 0.5 | 30 | 36 | 77.59±1.58 |
| 18 | 0.8 | 4: 6 | 1: 0.7 | 35 | 24 | 88.93±1.88 |
| K_1_ | 85.52 | 85.71 | 83.42 | 84.25 | 86.07 |  |
| K_2_ | 85.14 | 85.13 | 83.62 | 83.87 | 87.39 |  |
| K_3_ | 85.54 | 85.53 | 83.54 | 84.62 | 84.80 |  |
| R | 0.41 | 0.58 | 0.20 | 0.75 | 2.59 |  |
| Ranking of factors affecting DPPH radical scavenging activity of FHRB: E>D>B>A>C. Theoretical optimal combination of FHRB: A_3_B_1_C_2_D_2_E_2_. Inoculation quantity was 0.8‰, *B. subtilis*: *L. plantarum* = 2: 8, solid-liquid ratio was 1: 0.6, fermentation temperature was 35℃, fermentation time was 36 hours. | | | | | | |

**Table** S**2 Statistics of sequencing quantity for each sample**

| Sample ID | Input | Filtered | Denoised | Merged | Non-chimeric | Non-singleton |
| --- | --- | --- | --- | --- | --- | --- |
| 2.5%HRB1 | 116348 | 102095 | 97846 | 79423 | 56731 | 54720 |
| 2.5%HRB2 | 143088 | 128406 | 124176 | 101595 | 65642 | 62812 |
| 2.5%HRB3 | 118927 | 106157 | 102386 | 84818 | 56282 | 54261 |
| 2.5%HRB4 | 116666 | 103184 | 99439 | 81514 | 53823 | 51201 |
| 2.5%HRB5 | 110138 | 98463 | 94813 | 77872 | 51458 | 49506 |
| 2.5%HRB6 | 136714 | 120102 | 115912 | 95984 | 67926 | 65597 |
| 2.5%HRB7 | 116388 | 104461 | 100710 | 82479 | 56765 | 54796 |
| 2.5%HRB8 | 124420 | 110119 | 106229 | 84783 | 58917 | 57080 |
| 5.0%HRB1 | 132449 | 117207 | 113175 | 90404 | 65285 | 62533 |
| 5.0%HRB2 | 147658 | 131317 | 126904 | 105931 | 66997 | 64180 |
| 5.0%HRB3 | 145243 | 130237 | 125970 | 103111 | 67451 | 65212 |
| 5.0%HRB4 | 134510 | 117813 | 113536 | 88191 | 58982 | 56187 |
| 5.0%HRB5 | 142198 | 124031 | 118869 | 91228 | 57539 | 53550 |
| 5.0%HRB6 | 146513 | 129460 | 125936 | 107808 | 70268 | 67853 |
| 5.0%HRB7 | 117636 | 105434 | 101464 | 80705 | 52677 | 50460 |
| 5.0%HRB8 | 133284 | 117752 | 114042 | 93224 | 64805 | 62678 |
| 2.5%FHRB1 | 87511 | 76091 | 72644 | 54920 | 39161 | 37248 |
| 2.5%FHRB2 | 109720 | 96610 | 92890 | 73237 | 50491 | 48145 |
| 2.5%FHRB3 | 110620 | 97868 | 93944 | 74694 | 51721 | 49243 |
| 2.5%FHRB4 | 117185 | 102948 | 99550 | 80683 | 57021 | 55488 |
| 2.5%FHRB5 | 139309 | 122514 | 117889 | 93089 | 62311 | 59540 |
| 2.5%FHRB6 | 136958 | 121058 | 116559 | 92313 | 63815 | 61496 |
| 2.5%FHRB7 | 142078 | 127081 | 122447 | 96370 | 62915 | 59606 |
| 2.5%FHRB8 | 140630 | 123033 | 118894 | 96432 | 61925 | 59612 |
| 5.0%FHRB1 | 138026 | 122997 | 118271 | 94521 | 66640 | 63889 |
| 5.0%FHRB2 | 134759 | 118633 | 115223 | 96259 | 66418 | 64277 |
| 5.0%FHRB3 | 135951 | 121421 | 117632 | 97542 | 73087 | 71145 |
| 5.0%FHRB4 | 111261 | 97259 | 93318 | 72506 | 50715 | 48898 |
| 5.0%FHRB5 | 125456 | 110134 | 105771 | 81454 | 53128 | 49930 |
| 5.0%FHRB6 | 146843 | 129701 | 125383 | 103169 | 65627 | 62506 |
| 5.0%FHRB7 | 87149 | 76967 | 73886 | 60069 | 44680 | 43306 |
| 5.0%FHRB8 | 93634 | 81417 | 78547 | 63228 | 45450 | 44067 |

**Table S3 Relative abundances of taxon at the phylum and genus level**

| Taxon at the phylum level (%) | 2.5% HRB | 2.5% FHRB | 5.0% HRB | 5.0% FHRB |
| --- | --- | --- | --- | --- |
| Firmicutes | 50.22 | 55.89 | 56.83 | 53.37 |
| Bacteroidetes | 38.47 | 34.07 | 36.09 | 37.25 |
| Proteobacteria | 3.19 | 3.32 | 2.62 | 3.08 |
| Actinobacteria | 1.64 | 1.49 | 1.34 | 1.47 |
| Verrucomicrobia | 0.19 | 0.97 | 0.68 | 0.51 |
| Synergistetes | 0.49 | 0.60 | 0.34 | 0.37 |
| Tenericutes | 0.11 | 0.24 | 0.10 | 0.13 |
| Deferribacteres | 0.08 | 0.22 | 0.09 | 0.15 |
| Elusimicrobia | 0.08 | 0.15 | 0.11 | 0.13 |
| Fusobacteria | 0.02 | 0.12 | 0.09 | 0.10 |
| Taxon at the genus level (%) | 2.5% HRB | 2.5% FHRB | 5.0% HRB | 5.0% FHRB |
| Bacteroides | 23.58 | 21.91 | 20.32 | 22.42 |
| Ruminococcus | 13.81 | 11.08 | 15.60 | 15.20 |
| Lachnospira | 8.52 | 6.92 | 8.57 | 7.53 |
| Lactobacillus | 6.41 | 6.64 | 6.44 | 7.68 |
| Clostridiales | 6.36 | 5.45 | 5.25 | 4.02 |
| Faecalibacterium | 3.92 | 5.62 | 5.24 | 4.95 |
| Peptococcus | 3.24 | 1.43 | 1.42 | 2.73 |
| Prevotella | 2.14 | 2.84 | 1.43 | 1.30 |
| Paraprevotella | 1.69 | 2.37 | 1.52 | 1.61 |
| Oscillospira | 1.73 | 1.45 | 1.97 | 1.93 |
| Megamonas | 1.97 | 1.89 | 0.59 | 1.23 |
| Coprococcus | 1.69 | 1.16 | 2.23 | 1.56 |
| Blautia | 1.40 | 1.23 | 1.07 | 1.56 |
| Desulfovibrio | 1.35 | 1.05 | 1.16 | 1.06 |
| Phascolarctobacterium | 1.04 | 0.64 | 1.12 | 1.07 |
| S24-7 | 1.33 | 1.24 | 0.73 | 0.95 |
| Paraprevotellaceae | 2.37 | 1.52 | 1.61 | 1.69 |
| Peptostreptococcaceae | 0.35 | 0.46 | 1.38 | 1.80 |
| Phascolarctobacterium | 0.64 | 1.12 | 1.07 | 1.04 |
| Ruminococcus | 0.69 | 0.90 | 0.76 | 0.91 |
| Parabacteroides | 0.50 | 0.87 | 0.64 | 0.86 |
| Coriobacteriaceae | 0.70 | 0.77 | 0.55 | 0.45 |
| Paraprevotella | 0.24 | 0.44 | 0.92 | 0.73 |
| Turicibacter | 0.20 | 0.35 | 0.80 | 0.98 |
| Akkermansia | 0.19 | 0.96 | 0.68 | 0.49 |
| Subdoligranulum | 0.53 | 0.35 | 0.83 | 0.44 |
| Sutterella | 0.46 | 0.56 | 0.32 | 0.46 |
| Synergistaceae | 0.48 | 0.60 | 0.34 | 0.36 |
| Mogibacteriaceae | 0.32 | 0.43 | 0.39 | 0.27 |
| Butyricicoccus | 0.18 | 0.51 | 0.33 | 0.34 |

**Table** S**4 Differential metabolites identified between the two groups**

| Metabolites | *P*-value | VIP | FC **(2.5%FHRB /2.5%HRB)** | Metabolites | *P*-value | VIP | FC **(5.0%HRB /2.5%HRB)** |
| --- | --- | --- | --- | --- | --- | --- | --- |
| Triethylamine | 0.028 | 1.70 | 0.33 | p-Cresol | 0.028 | 1.68 | 2.03 |
| 1-Aminocyclopropanecarboxylic acid | 0.010 | 1.87 | 1.94 | 1,2,3-Trihydroxybenzene | 0.049 | 1.57 | 1.44 |
| Choline | 0.007 | 1.39 | 4.81 | Isochavicol | 0.021 | 2.19 | 0.57 |
| 2-Phenylethanol | 0.028 | 1.60 | 0.70 | Indole-3-carboxylic acid | 0.015 | 2.48 | 0.34 |
| m-Cresol | 0.021 | 2.02 | 0.27 | Safrole | 0.015 | 2.47 | 0.30 |
| Glutarate semialdehyde | 0.015 | 1.47 | 0.72 | 5-Hydroxylysine | 0.021 | 2.13 | 1.11 |
| Indole | 0.048 | 1.02 | 1.14 | Pterin | 0.049 | 1.88 | 0.39 |
| 2,6-Dimethylaniline | 0.038 | 1.23 | 0.74 | S-Carboxymethyl-L-cysteine | 0.038 | 1.86 | 0.37 |
| N-Acetylputrescine | 0.049 | 1.41 | 1.91 | Thiabendazole | 0.007 | 2.41 | 1.32 |
| L-Leucine | 0.002 | 1.84 | 2.26 | Spermine | 0.003 | 2.18 | 1.21 |
| Creatine | 0.015 | 1.48 | 1.80 | 5-Methoxyindoleacetate | 0.010 | 2.19 | 0.43 |
| Ureidopropionic acid | 0.049 | 1.58 | 1.97 | Prephenate | 0.049 | 1.38 | 0.67 |
| Cinnamaldehyde | 0.010 | 1.81 | 0.56 | N-Acetyl-L-2-amino-6-oxopimelate | 0.007 | 1.77 | 1.68 |
| 1,3-Dihydro-(2H)-indol-2-one | 0.028 | 1.55 | 0.59 | Fenfluramine | 0.021 | 2.30 | 0.24 |
| Isochavicol | 0.007 | 1.86 | 0.52 | N(omega)-Nitro-L-arginine methyl ester | 0.049 | 2.31 | 1.89 |
| Pulegone | 0.038 | 1.54 | 0.66 | Confertifolin | 0.015 | 2.11 | 2.55 |
| Phenylacetic acid | 0.021 | 1.76 | 1.27 | Linoleic acid | 0.038 | 2.20 | 2.39 |
| Acetylphosphate | 0.001 | 2.22 | 1.23 | 15-Deoxy-d-12,14-PGJ2 | 0.010 | 1.93 | 0.77 |
| 2-Naphthylamine | 0.021 | 1.42 | 0.51 | (R)-10-Hydroxystearate | 0.010 | 2.10 | 2.03 |
| 2-Dehydropantoate | 0.049 | 1.61 | 0.36 | Allopregnanolone | 0.021 | 2.19 | 2.08 |
| L-Glutamine | 0.028 | 1.69 | 0.52 | N, N-Dimethylsphing-4-enine | 0.028 | 1.54 | 2.24 |
| L-Methionine | 0.038 | 1.53 | 1.37 | all-trans-5,6-Epoxyretinoic acid | 0.049 | 1.89 | 0.68 |
| Metaraminol | 0.003 | 1.80 | 0.54 | Labetalol | 0.049 | 2.01 | 0.64 |
| D-Cathinone | 0.005 | 1.90 | 0.50 | Carnosol | 0.038 | 2.27 | 0.47 |
| D-Arabitol | 0.038 | 1.47 | 0.72 | 12-Keto-tetrahydro-leukotriene B4 | 0.010 | 2.47 | 0.49 |
| 3D-3,5_4-Trihydroxycyclohexane-1,2-dione | 0.021 | 1.67 | 0.47 | 6-Keto-prostaglandin F1a | 0.001 | 2.57 | 2.79 |
| 4-Hydroxy-2-quinolone | 0.049 | 1.55 | 0.73 | Maslinic acid | 0.028 | 2.22 | 0.28 |
| 5-Hydroxylysine | 0.002 | 2.18 | 1.17 | Soyasaponin I | 0.021 | 1.83 | 4.11 |
| Normetanephrine | 0.021 | 1.49 | 1.78 | Phenylacetaldehyde | 0.015 | 2.09 | 1.66 |
| 2,4,6_3,5-Pentahydroxycyclohexanone | 0.049 | 1.66 | 0.59 | Purine | 0.003 | 2.78 | 3.30 |
| S-Carboxymethyl-L-cysteine | 0.007 | 1.94 | 0.41 | N-Acetylornithine | 0.049 | 1.77 | 0.62 |
| 4-Acetamidobenzoic acid | 0.015 | 1.82 | 0.55 | Shikimic acid | 0.010 | 2.26 | 0.60 |
| Beta-Tyrosine | 0.015 | 1.87 | 0.65 | Hippuric acid | 0.028 | 2.69 | 1.41 |
| Phosphorylcholine | 0.038 | 1.78 | 0.58 | 4-Hydroxyphenylpyruvic acid | 0.049 | 2.11 | 1.78 |
| N-Alpha-acetyllysine | 0.049 | 1.58 | 0.70 | L-Tryptophan | 0.038 | 1.96 | 0.50 |
| 2-Keto-6-acetamidocaproate | 0.049 | 1.62 | 0.49 | Adenosine diphosphate ribose | 0.021 | 2.02 | 3.19 |
| Homocitrulline | 0.010 | 1.85 | 0.59 | Metabolites | *P*-value | VIP | FC **(5.0%FHRB /2.5%FHRB)** |
| Glycylleucine | 0.001 | 2.47 | 3.23 | Cytosine | 0.049 | 1.03 | 1.67 |
| meso-2,6-Diaminoheptanedioate | 0.038 | 1.40 | 0.61 | L-Proline | 0.015 | 1.51 | 1.69 |
| (-)-Jasmonic acid | 0.028 | 1.61 | 0.51 | 2,6-Dimethylaniline | 0.021 | 1.69 | 1.45 |
| 2-Amino-2-deoxy-D-gluconate | 0.028 | 1.74 | 0.69 | Agmatine | 0.015 | 1.72 | 0.40 |
| Thiabendazole | 0.005 | 1.81 | 1.26 | Pulegone | 0.021 | 1.69 | 1.61 |
| O-Acetylcarnitine | 0.005 | 1.88 | 0.40 | 4-Hydroxyphenylacetaldehyde | 0.038 | 1.75 | 0.58 |
| 5-Methoxyindoleacetate | 0.007 | 1.86 | 0.41 | Methylimidazoleacetic acid | 0.010 | 1.66 | 2.37 |
| N-Acetyl-D-phenylalanine | 0.028 | 1.40 | 0.35 | 2,3-Dihydroxy-3-methylvalerate | 0.002 | 1.66 | 1.66 |
| Neostigmine | 0.021 | 1.20 | 0.54 | N-Methyltyramine | 0.049 | 1.33 | 1.58 |
| Hydroxykynurenine | 0.021 | 1.94 | 0.58 | p-Octopamine | 0.028 | 1.12 | 1.06 |
| Prephenate | 0.003 | 1.93 | 0.67 | N-Acetylhistamine | 0.049 | 1.33 | 1.27 |
| N-Acetyl-L-2-amino-6-oxopimelate | 0.007 | 1.62 | 1.47 | L-Histidine | 0.049 | 1.31 | 1.40 |
| Fenfluramine | 0.049 | 1.52 | 0.44 | 4-Acetamido-2-aminobutanoic acid | 0.038 | 1.51 | 0.65 |
| N2-Succinyl-L-ornithine | 0.021 | 1.78 | 1.65 | 3D-3,5_4-Trihydroxycyclohexane-1,2-dione | 0.038 | 1.41 | 1.93 |
| Costunolide | 0.015 | 1.88 | 0.43 | 4-Hydroxy-2-quinolone | 0.002 | 2.03 | 1.63 |
| D-Octopine | 0.038 | 1.51 | 1.59 | L-Homophenylalanine | 0.049 | 1.53 | 1.35 |
| Pyridoxal 5'-phosphate | 0.002 | 2.06 | 0.63 | 2-Deoxystreptamine | 0.015 | 1.71 | 1.38 |
| Juvenile hormone III | 0.049 | 1.54 | 1.68 | L-Methionine S-oxide | 0.049 | 1.48 | 0.40 |
| Rivastigmine | 0.007 | 2.25 | 0.21 | Nalpha-Methylhistidine | 0.015 | 1.32 | 1.64 |
| Estrone | 0.028 | 1.51 | 0.66 | Indole-3-acetate | 0.049 | 1.29 | 0.50 |
| Genistein | 0.028 | 1.67 | 0.71 | 4-Acetamidobenzoic acid | 0.010 | 1.66 | 1.81 |
| Retinal | <0.001 | 2.45 | 0.09 | 4-Pyridoxic acid | 0.002 | 1.95 | 1.51 |
| 6beta-Hydroxytestosterone | 0.015 | 1.53 | 5.80 | Phosphoserine | 0.028 | 1.68 | 1.29 |
| Phenyllactic acid | <0.001 | 2.23 | 0.32 | 5-Guanidino-3-methyl-2-oxopentanoate | 0.028 | 1.27 | 0.71 |
| Adrenosterone | 0.049 | 1.62 | 0.53 | (2S,4S)-4-Hydroxy-2,3,4,5-tetrahydrodipicolinate | 0.038 | 1.49 | 1.56 |
| Allopregnanolone | 0.010 | 1.82 | 2.42 | N-Alpha-acetyllysine | 0.010 | 1.85 | 1.55 |
| Sphinganine | 0.028 | 1.61 | 0.47 | Homocitrulline | 0.005 | 1.73 | 1.50 |
| Dihydrocapsaicin | 0.049 | 1.46 | 0.60 | meso-2,6-Diaminoheptanedioate | 0.049 | 1.37 | 1.57 |
| Praziquantel | 0.049 | 1.28 | 3.19 | Neocnidilide | 0.007 | 1.51 | 1.94 |
| all-trans-5,6-Epoxyretinoic acid | 0.003 | 2.12 | 0.55 | 2-Amino-2-deoxy-D-gluconate | 0.001 | 2.08 | 1.62 |
| Ubiquinone-1 | 0.038 | 1.46 | 0.73 | L-Dopa | 0.010 | 1.83 | 1.84 |
| 5,6-DHET | 0.049 | 1.61 | 0.49 | N-Acetylhistidine | 0.005 | 1.76 | 1.80 |
| Citalopram | 0.049 | 1.54 | 0.67 | gamma-Glutamyl-beta-aminopropiononitrile | 0.038 | 1.53 | 1.77 |
| 2,3-Dinor-8-iso prostaglandin F2alpha | 0.021 | 1.35 | 0.39 | Dodecanoic acid | 0.038 | 1.62 | 1.60 |
| 17beta-Acetamidoandrost-4-en-3-one | 0.038 | 1.58 | 6.12 | N-Acetyl-D-galactosamine | 0.010 | 1.81 | 1.61 |
| Labetalol | 0.038 | 1.56 | 0.62 | N6-Acetyl-N6-hydroxy-L-lysine | 0.049 | 1.40 | 0.38 |
| Kyotorphin | 0.015 | 1.65 | 0.59 | Isoelemicin | <0.001 | 2.10 | 1.62 |
| 17alpha,21-Dihydroxypregnenolone | 0.021 | 1.57 | 0.69 | Pilocarpine | 0.049 | 1.55 | 1.53 |
| Misoprostol | 0.007 | 1.68 | 0.42 | Harmine | 0.015 | 1.40 | 1.63 |
| Resolvin D2 | 0.005 | 1.99 | 0.28 | N-a-Acetylcitrulline | 0.010 | 1.44 | 1.70 |
| N-Acetyllactosamine | 0.021 | 1.69 | 0.38 | N-Acetylserotonin | <0.001 | 1.99 | 2.12 |
| 6-Keto-prostaglandin F1a | 0.007 | 1.96 | 2.42 | Prephenate | 0.015 | 1.79 | 1.31 |
| Lovastatin | 0.028 | 1.55 | 0.49 | Deoxyuridine | 0.015 | 1.66 | 1.57 |
| Allocholic acid | 0.038 | 1.35 | 0.66 | Trioxsalen | 0.038 | 1.47 | 1.40 |
| LysoPA(16_0_0_0) | 0.015 | 1.69 | 0.43 | Traumatic Acid | 0.015 | 1.53 | 1.69 |
| Licoricidin | 0.007 | 2.01 | 3.41 | Propazine | 0.038 | 1.39 | 1.93 |
| alpha-Tocopherol | <0.001 | 2.65 | 0.16 | Costunolide | 0.010 | 1.55 | 1.90 |
| Hecogenin | 0.021 | 1.80 | 0.19 | N-[(2S)-2-Amino-2-carboxyethyl]-L-glutamate | 0.003 | 1.91 | 1.53 |
| 16-Feruloyloxypalmitate | 0.021 | 1.68 | 0.46 | Equol | 0.028 | 1.55 | 1.66 |
| N1,N8-Bis(4-coumaroyl)spermidine | 0.041 | 1.25 | 0.18 | Ribavirin | 0.038 | 1.44 | 1.45 |
| Maslinic acid | 0.038 | 1.76 | 0.46 | beta-Alanyl-L-arginine | 0.002 | 1.95 | 1.57 |
| alpha-D-Galactosyl-1,3-beta-D-galactosyl-1,4-N-acetyl-D-glucosamine | 0.003 | 1.93 | 0.20 | Pyridoxamine 5'-phosphate | 0.007 | 1.74 | 1.52 |
| NAD | 0.001 | 2.32 | 0.19 | 6-Hydroxymelatonin | 0.038 | 1.59 | 1.56 |
| Succinic acid semialdehyde | 0.021 | 1.63 | 2.88 | Deoxyadenosine | 0.015 | 1.57 | 1.34 |
| alpha-Ketoisovaleric acid | 0.049 | 1.66 | 2.15 | 16-Hydroxy hexadecanoic acid | 0.010 | 1.71 | 1.55 |
| 5-Aminopentanoic acid | 0.028 | 1.77 | 1.48 | Imidacloprid | <0.001 | 1.69 | 3.13 |
| Purine | 0.028 | 1.61 | 1.96 | Parthenin | 0.038 | 1.39 | 1.59 |
| L-Cysteine | 0.001 | 2.49 | 0.44 | Linoleic acid | 0.038 | 1.59 | 1.81 |
| 2-Dehydro-3-deoxy-D-xylonate | 0.021 | 1.70 | 2.68 | Tramadol | 0.005 | 1.84 | 1.80 |
| D-Ribose | 0.015 | 1.86 | 1.35 | Thiamine | 0.015 | 1.23 | 2.20 |
| 3,4-Dihydroxybenzoate | 0.049 | 1.83 | 3.46 | Nevirapine | 0.015 | 1.50 | 1.89 |
| Citrulline | 0.021 | 1.72 | 0.56 | Androstenedione | 0.010 | 1.59 | 1.50 |
| L-Tryptophan | 0.038 | 1.68 | 0.46 | Estrone | 0.015 | 1.55 | 1.57 |
| Indolelactic acid | 0.007 | 2.11 | 0.26 | Nordiazepam | 0.005 | 1.72 | 2.17 |
| N-Acetyl-L-phenylalanine | 0.015 | 1.77 | 0.38 | Genistein | 0.001 | 2.17 | 1.83 |
| Citrinin | 0.007 | 1.91 | 0.60 | Sotalol | 0.028 | 1.19 | 1.79 |
| N2-gamma-Glutamylglutamine | 0.028 | 1.41 | 3.00 | N2-Succinyl-L-arginine | 0.028 | 1.38 | 1.44 |
| 9,10-DHOME | 0.005 | 1.95 | 1.94 | Saccharopine | 0.038 | 1.45 | 1.77 |
| 6-Ketoprostaglandin E1 | 0.038 | 1.69 | 0.58 | 2-Cyano-3,3-diphenylacrylic acid ethyl ester | 0.049 | 1.17 | 1.41 |
| FMN | 0.028 | 1.83 | 0.21 | 1-Methyladenosine | 0.003 | 1.99 | 1.51 |
| Adenosine diphosphate ribose | 0.049 | 1.39 | 2.66 | Oleamide | 0.015 | 1.34 | 1.80 |
| Metabolites | *P*-value | VIP | FC **(5.0%FHRB /5.0%HRB)** | Norcodeine | 0.005 | 1.63 | 1.76 |
| L-Leucine | 0.028 | 1.53 | 0.71 | Piperine | 0.028 | 1.59 | 1.50 |
| Hypoxanthine | 0.038 | 1.73 | 0.67 | N1,N12-Diacetylspermine | 0.007 | 1.77 | 1.86 |
| Methylimidazoleacetic acid | 0.015 | 2.20 | 0.33 | Anastrozole | 0.049 | 1.36 | 1.47 |
| Pimelate | 0.049 | 1.94 | 1.89 | Trimeprazine | 0.049 | 1.47 | 1.42 |
| Phosphorylcholine | 0.002 | 2.40 | 1.59 | Sphingosine | 0.021 | 1.51 | 1.79 |
| Acetylphosphate | <0.001 | 2.01 | 0.80 | Adrenosterone | 0.021 | 1.37 | 1.66 |
| O-Acetylcarnitine | 0.038 | 1.83 | 1.60 | Sphinganine | 0.049 | 1.37 | 1.75 |
| Cytosine | 0.049 | 1.54 | 0.48 | Nicotianamine | 0.028 | 1.36 | 1.40 |
| Biocytin | 0.038 | 1.71 | 0.61 | 8,9-EET | 0.007 | 1.65 | 1.76 |
| Thiamine | 0.015 | 1.93 | 0.43 | 4'-Oxolividamine | 0.038 | 1.55 | 1.79 |
| N-Acetylleucine | 0.021 | 2.11 | 0.34 | Clomipramine | 0.021 | 1.52 | 2.24 |
| Thymine | 0.038 | 1.41 | 0.52 | Ubiquinone-1 | 0.002 | 1.96 | 1.62 |
| Saccharopine | 0.003 | 1.92 | 0.35 | Citalopram | <0.001 | 2.10 | 1.90 |
| Nalpha-Methylhistidine | 0.010 | 2.04 | 0.57 | 2alpha-Methylpregn-4-ene-3,20-dione | 0.028 | 1.10 | 1.67 |
| Agmatine | 0.038 | 1.81 | 2.07 | Hydroxychloroquine | 0.015 | 1.24 | 1.66 |
| Pyrophosphate | 0.049 | 1.90 | 0.63 | 12-Keto-tetrahydro-leukotriene B4 | 0.049 | 1.48 | 0.72 |
| Alpha-Tocotrienol | 0.028 | 1.76 | 0.55 | Kyotorphin | 0.003 | 1.84 | 1.66 |
| N1,N8-Bis(4-coumaroyl)spermidine | 0.001 | 2.63 | 4.96 | Erucic acid | 0.028 | 1.51 | 0.81 |
| 3-Keto-4-methylzymosterol | 0.015 | 1.76 | 2.83 | Bioresmethrin | 0.049 | 1.41 | 1.49 |
| Hecogenin | <0.001 | 2.54 | 5.67 | Naltrexone | 0.007 | 1.68 | 1.63 |
| p-Octopamine | 0.010 | 2.28 | 0.94 | Coniferin | 0.007 | 1.79 | 2.46 |
| Hydroquinone | 0.028 | 1.85 | 0.66 | L-Alanyl-gamma-D-glutamyl-L-lysine | 0.010 | 1.96 | 3.20 |
| alpha-Tocopherol | 0.005 | 2.40 | 4.79 | N-Acetylmuramoyl-Ala | 0.038 | 1.13 | 1.33 |
| Phenylacetaldehyde | 0.038 | 1.88 | 1.64 | 17alpha,21-Dihydroxypregnenolone | 0.021 | 1.67 | 1.60 |
| Retinal | <0.001 | 3.18 | 48.27 | Prostaglandin I2 | 0.005 | 1.85 | 2.28 |
| Phenyllactic acid | <0.001 | 2.53 | 2.43 | Vincamine | 0.021 | 1.45 | 2.39 |
| 19-Hydroxytestosterone | 0.021 | 1.82 | 0.51 | Yohimbine | 0.028 | 1.60 | 1.50 |
| 5-Nitro-2-benzoic acid | 0.038 | 1.71 | 0.55 | Nitrendipine | 0.005 | 1.80 | 1.51 |
| 2-Dehydro-3-deoxy-L-rhamnonate | 0.049 | 1.72 | 0.69 | 1-Arachidonoylglycerol | 0.021 | 1.58 | 0.63 |
| Metaraminol | 0.038 | 1.87 | 1.29 | Chelirubine | 0.038 | 1.38 | 0.49 |
| Caprylic acid | 0.038 | 1.97 | 1.96 | Enalapril | 0.028 | 1.44 | 1.41 |
| Coniferin | 0.049 | 1.82 | 0.40 | S-Inosyl-L-homocysteine | 0.001 | 2.25 | 2.08 |
| Cinnamaldehyde | 0.038 | 1.64 | 1.44 | beta-Sitosterol | 0.010 | 1.78 | 0.47 |
| 4-Methoxy-2,2'-bipyrrole-5-carbaldehyde | 0.015 | 1.83 | 0.33 | Licoricidin | 0.038 | 1.51 | 0.38 |
| Neocnidilide | 0.001 | 2.47 | 0.44 | Copal-8-ol diphosphate | 0.015 | 1.55 | 1.70 |
| Purine | 0.015 | 2.37 | 2.58 | Protoporphyrin IX | 0.021 | 1.16 | 0.42 |
| alpha-D-Galactosyl-1,3-beta-D-galactosyl-1,4-N-acetyl-D-glucosamine | <0.001 | 2.87 | 9.44 | (R)-2,3-Dihydroxy-isovalerate | 0.038 | 1.22 | 1.82 |
| 6-Methoxymellein | 0.038 | 1.86 | 0.31 | L-Cysteine | 0.038 | 1.59 | 1.48 |
| Xanthosine | 0.015 | 2.09 | 2.00 | L-Aspartic acid | 0.010 | 1.96 | 0.29 |
| Prostaglandin I2 | 0.038 | 1.98 | 0.45 | L-Ribulose | 0.028 | 1.62 | 1.33 |
| Hippuric acid | 0.021 | 2.11 | 1.37 | Uric acid | 0.010 | 1.91 | 0.58 |
| Resolvin D2 | <0.001 | 2.85 | 6.21 | myo-Inositol | 0.010 | 1.82 | 2.34 |
| Pseudouridine | 0.049 | 1.61 | 0.57 | D-Mannose | 0.007 | 2.28 | 1.67 |
| delta-Tocotrienol | 0.002 | 2.46 | 0.18 | Cysteine-S-sulfate | 0.049[ | 1.73 | 1.58 |
| Dethiobiotin | 0.015 | 2.11 | 0.42 | Pantothenic acid | 0.021 | 2.02 | 2.01 |
| 5'-S-Methyl-5'-thioinosine | 0.005 | 2.44 | 0.52 | Thymidine | <0.001 | 2.76 | 1.80 |
| o-Cresol | <0.001 | 2.04 | 0.53 | Pentadecanoic acid | 0.049 | 1.58 | 1.85 |
|  |  |  |  | Arbutin | 0.015 | 1.84 | 1.74 |
|  |  |  |  | N2-gamma-Glutamylglutamine | 0.049 | 1.47 | 0.38 |
|  |  |  |  | 5'-S-Methyl-5'-thioinosine | 0.002 | 2.28 | 2.10 |
|  |  |  |  | Chenodeoxycholic acid | 0.021 | 1.92 | 1.66 |

**Table** S**5 Enrichment analysis of differentially expressed metabolites in cecal chyme**

| Metabolic pathways between 2.5% HRB and 2.5% FHRB groups | | | |
| --- | --- | --- | --- |
| Pathway name | Hits | *P-*value | Impact |
| Oxidative phosphorylation | 3 | 0.016 | 0.15 |
| Pantothenate and CoA biosynthesis | 4 | 0.019 | 0.11 |
| Aminoacyl-tRNA biosynthesis | 5 | 0.032 | 0.10 |
| Phenylalanine metabolism | 5 | 0.055 | 0.08 |
| Vitamin B6 metabolism | 3 | 0.077 | 0.13 |
| Thiamine metabolism | 3 | 0.090 | 0.11 |
| Lysine degradation | 4 | 0.093 | 0.03 |
| Glycine, serine and threonine metabolism | 4 | 0.093 | 0.05 |
| Phenylalanine, tyrosine and tryptophan biosynthesis | 3 | 0.111 | 0.08 |
| Arginine and proline metabolism | 5 | 0.131 | 0.06 |
| mTOR signaling pathway | 1 | 0.131 | 0.25 |
| Taurine and hypotaurine metabolism | 2 | 0.175 | 0.09 |
| Valine, leucine and isoleucine biosynthesis | 2 | 0.188 | 0.13 |
| Arginine biosynthesis | 2 | 0.188 | 0.15 |
| Retinol metabolism | 2 | 0.214 | 0.13 |
| Phototransduction | 1 | 0.246 | 0.10 |
| Alanine, aspartate and glutamate metabolism | 2 | 0.252 | 0.05 |
| Arachidonic acid metabolism | 4 | 0.260 | 0.03 |
| Ferroptosis | 2 | 0.265 | 0.09 |
| Sulfur relay system | 1 | 0.322 | 0.13 |
| Pentose phosphate pathway | 2 | 0.343 | 0.01 |
| D-Glutamine and D-glutamate metabolism | 1 | 0.368 | 0.10 |
| Cysteine and methionine metabolism | 3 | 0.373 | 0.13 |
| Valine, leucine and isoleucine degradation | 2 | 0.430 | 0.06 |
| Steroid hormone biosynthesis | 4 | 0.450 | 0.04 |
| Inositol phosphate metabolism | 2 | 0.489 | 0.00 |
| Nitrogen metabolism | 1 | 0.489 | 0.03 |
| Riboflavin metabolism | 1 | 0.507 | 0.18 |
| Tyrosine metabolism | 3 | 0.511 | 0.02 |
| ABC transporters | 5 | 0.525 | 0.04 |
| Glycerophospholipid metabolism | 2 | 0.543 | 0.08 |
| Tryptophan metabolism | 3 | 0.554 | 0.04 |
| Nicotinate and nicotinamide metabolism | 2 | 0.574 | 0.07 |
| Pentose and glucuronate interconversions | 2 | 0.584 | 0.02 |
| Sphingolipid metabolism | 1 | 0.587 | 0.05 |
| Linoleic acid metabolism | 1 | 0.629 | 0.03 |
| Pyrimidine metabolism | 2 | 0.666 | 0.03 |
| Pyruvate metabolism | 1 | 0.666 | 0.03 |
| beta-Alanine metabolism | 1 | 0.678 | 0.04 |
| Sulfur metabolism | 1 | 0.689 | 0.02 |
| Glutathione metabolism | 1 | 0.740 | 0.02 |
| Butanoate metabolism | 1 | 0.774 | 0.05 |
| alpha-Linolenic acid metabolism | 1 | 0.790 | 0.02 |
| Primary bile acid biosynthesis | 1 | 0.811 | 0.03 |
| Drug metabolism - cytochrome P450 | 2 | 0.812 | 0.03 |
| Ubiquinone and other terpenoid-quinone biosynthesis | 2 | 0.836 | 0.02 |
| Drug metabolism - other enzymes | 1 | 0.842 | 0.02 |
| Purine metabolism | 2 | 0.849 | 0.02 |
| Glyoxylate and dicarboxylate metabolism | 1 | 0.890 | 0.00 |
| Metabolic pathways between 5.0% HRB and 5.0% FHRB groups | | | |
| Pathway name | Hits | *P-*value | Impact |
| Biotin metabolism | 3 | 0.008 | 0.08 |
| mTOR signaling pathway | 1 | 0.059 | 0.25 |
| Pyrimidine metabolism | 3 | 0.073 | 0.01 |
| VEGF signaling pathway | 1 | 0.087 | 0.17 |
| Phototransduction | 1 | 0.114 | 0.10 |
| Sulfur relay system | 1 | 0.154 | 0.07 |
| Ubiquinone and other terpenoid-quinone biosynthesis | 3 | 0.160 | 0.02 |
| Lipoic acid metabolism | 1 | 0.179 | 0.08 |
| Neuroactive ligand-receptor interaction | 2 | 0.184 | 0.04 |
| Vascular smooth muscle contraction | 1 | 0.216 | 0.06 |
| Oxidative phosphorylation | 1 | 0.216 | 0.10 |
| Phenylalanine metabolism | 2 | 0.228 | 0.05 |
| Caffeine metabolism | 1 | 0.285 | 0.03 |
| Taurine and hypotaurine metabolism | 1 | 0.285 | 0.03 |
| Valine, leucine and isoleucine biosynthesis | 1 | 0.295 | 0.06 |
| Retinol metabolism | 1 | 0.317 | 0.12 |
| ABC transporters | 3 | 0.345 | 0.02 |
| Ferroptosis | 1 | 0.357 | 0.02 |
| Pyruvate metabolism | 1 | 0.377 | 0.03 |
| Thiamine metabolism | 1 | 0.377 | 0.07 |
| Purine metabolism | 2 | 0.422 | 0.04 |
| Valine, leucine and isoleucine degradation | 1 | 0.474 | 0.02 |
| Histidine metabolism | 1 | 0.513 | 0.04 |
| Lysine degradation | 1 | 0.535 | 0.05 |
| Glycerophospholipid metabolism | 1 | 0.549 | 0.01 |
| Aminoacyl-tRNA biosynthesis | 1 | 0.549 | 0.02 |
| Fructose and mannose metabolism | 1 | 0.562 | 0.01 |
| Fatty acid biosynthesis | 1 | 0.589 | 0.01 |
| Steroid biosynthesis | 1 | 0.589 | 0.02 |
| Cysteine and methionine metabolism | 1 | 0.619 | 0.00 |
| Arachidonic acid metabolism | 1 | 0.684 | 0.01 |
| Tyrosine metabolism | 1 | 0.698 | 0.00 |
| Arginine and proline metabolism | 1 | 0.698 | 0.01 |
| Steroid hormone biosynthesis | 1 | 0.783 | 0.00 |
| Metabolic pathways between 2.5% HRB and 5.0% HRB groups | | | |
| Pathway name | Hits | *P-*value | Impact |
| Phenylalanine, tyrosine and tryptophan biosynthesis | 4 | 0.001 | 0.13 |
| Arachidonic acid metabolism | 3 | 0.054 | 0.04 |
| PPAR signaling pathway | 1 | 0.056 | 0.20 |
| Phenylalanine metabolism | 2 | 0.151 | 0.05 |
| Arginine biosynthesis | 1 | 0.235 | 0.02 |
| Tryptophan metabolism | 2 | 0.248 | 0.04 |
| Retinol metabolism | 1 | 0.253 | 0.01 |
| Linoleic acid metabolism | 1 | 0.278 | 0.06 |
| Pantothenate and CoA biosynthesis | 1 | 0.295 | 0.01 |
| beta-Alanine metabolism | 1 | 0.311 | 0.03 |
| Glutathione metabolism | 1 | 0.358 | 0.01 |
| Glycine, serine and threonine metabolism | 1 | 0.443 | 0.00 |
| Lysine degradation | 1 | 0.443 | 0.01 |
| Aminoacyl-tRNA biosynthesis | 1 | 0.456 | 0.02 |
| Neuroactive ligand-receptor interaction | 1 | 0.456 | 0.02 |
| Biosynthesis of unsaturated fatty acids | 1 | 0.581 | 0.02 |
| Arginine and proline metabolism | 1 | 0.600 | 0.02 |
| Tyrosine metabolism | 1 | 0.600 | 0.03 |
| Ubiquinone and other terpenoid-quinone biosynthesis | 1 | 0.662 | 0.02 |
| Purine metabolism | 1 | 0.673 | 0.00 |
| Steroid hormone biosynthesis | 1 | 0.689 | 0.00 |
| Metabolic pathways between 2.5% FHRB and 5.0% FHRB groups | | | |
| Pathway name | Hits | *P-*value | Impact |
| Pantothenate and CoA biosynthesis | 4 | 0.028 | 0.14 |
| beta-Alanine metabolism | 4 | 0.035 | 0.16 |
| Cysteine and methionine metabolism | 6 | 0.036 | 0.11 |
| Aminoacyl-tRNA biosynthesis | 5 | 0.052 | 0.08 |
| Sulfur relay system | 2 | 0.067 | 0.20 |
| Histidine metabolism | 4 | 0.112 | 0.14 |
| Vascular smooth muscle contraction | 2 | 0.129 | 0.13 |
| Glycine, serine and threonine metabolism | 4 | 0.132 | 0.08 |
| Neuroactive ligand-receptor interaction | 4 | 0.147 | 0.08 |
| Lysosome | 1 | 0.149 | 0.25 |
| Apoptosis | 1 | 0.149 | 0.25 |
| ABC transporters | 8 | 0.175 | 0.06 |
| PPAR signaling pathway | 1 | 0.182 | 0.20 |
| VEGF signaling pathway | 1 | 0.215 | 0.17 |
| Valine, leucine and isoleucine biosynthesis | 2 | 0.229 | 0.06 |
| Arginine biosynthesis | 2 | 0.229 | 0.11 |
| Sphingolipid metabolism | 2 | 0.259 | 0.12 |
| Galactose metabolism | 3 | 0.271 | 0.02 |
| Vitamin B6 metabolism | 2 | 0.318 | 0.10 |
| Necroptosis | 1 | 0.331 | 0.08 |
| Thiamine metabolism | 2 | 0.347 | 0.13 |
| C-type lectin receptor signaling pathway | 1 | 0.383 | 0.08 |
| Phenylalanine, tyrosine and tryptophan biosynthesis | 2 | 0.390 | 0.05 |
| Oxidative phosphorylation | 1 | 0.475 | 0.05 |
| Pyrimidine metabolism | 3 | 0.476 | 0.04 |
| Steroid hormone biosynthesis | 4 | 0.554 | 0.08 |
| Inositol phosphate metabolism | 2 | 0.559 | 0.04 |
| Arachidonic acid metabolism | 3 | 0.574 | 0.04 |
| Taurine and hypotaurine metabolism | 1 | 0.588 | 0.06 |
| Arginine and proline metabolism | 3 | 0.601 | 0.03 |
| Tyrosine metabolism | 3 | 0.601 | 0.07 |
| Tryptophan metabolism | 3 | 0.643 | 0.07 |
| Drug metabolism - cytochrome P450 | 3 | 0.675 | 0.03 |
| Linoleic acid metabolism | 1 | 0.677 | 0.06 |
| Alanine, aspartate and glutamate metabolism | 1 | 0.677 | 0.15 |
| Phosphatidylinositol signaling system | 1 | 0.690 | 0.02 |
| Ferroptosis | 1 | 0.690 | 0.07 |
| Glycolysis / Gluconeogenesis | 1 | 0.714 | 0.00 |
| Sulfur metabolism | 1 | 0.737 | 0.02 |
| Pentose phosphate pathway | 1 | 0.757 | 0.01 |
| Glutathione metabolism | 1 | 0.785 | 0.02 |
| Biosynthesis of unsaturated fatty acids | 2 | 0.798 | 0.03 |
| alpha-Linolenic acid metabolism | 1 | 0.832 | 0.02 |
| Neomycin, kanamycin and gentamicin biosynthesis | 2 | 0.837 | 0.02 |
| Primary bile acid biosynthesis | 1 | 0.851 | 0.03 |
| Ascorbate and aldarate metabolism | 1 | 0.868 | 0.03 |
| Lysine degradation | 1 | 0.868 | 0.05 |
| Fructose and mannose metabolism | 1 | 0.888 | 0.04 |
| Nicotinate and nicotinamide metabolism | 1 | 0.893 | 0.02 |
| Purine metabolism | 2 | 0.896 | 0.02 |
| Pentose and glucuronate interconversions | 1 | 0.897 | 0.07 |
| Fatty acid biosynthesis | 1 | 0.905 | 0.01 |
| Steroid biosynthesis | 1 | 0.905 | 0.02 |
| Ubiquinone and other terpenoid-quinone biosynthesis | 1 | 0.977 | 0.01 |
| Amino sugar and nucleotide sugar metabolism | 1 | 0.988 | 0.01 |
| Porphyrin and chlorophyll metabolism | 1 | 0.997 | 0.02 |

Notes: Hits, number of overall differential metabolites in target metabolic pathways; *P*-value, of hypergeometric distribution test, smaller represents more significant impact of detected differential metabolites on the pathway; impact, metabolic pathway impact value, the larger the effect of detected differential metabolites on the target pathway.
